# Shifting Merocyanine-Imine Exchange with Visible Light

**DOI:** 10.1021/jacs.5c17606

**Published:** 2026-02-05

**Authors:** Alwin Drichel, Stefan Hecht

**Affiliations:** † 38813 DWI-Leibniz Institute for Interactive Materials, 52074 Aachen, Germany; ‡ Institute of Technical and Macromolecular Chemistry, RWTH Aachen University, 52074 Aachen, Germany; § Department of Chemistry & Center for the Science of Materials Berlin, 9373 Humboldt-Universität zu Berlin, 12489 Berlin, Germany

## Abstract

We show the dynamic covalent exchange between merocyanines and imines and demonstrate how the equilibrium composition can be shifted with visible light. For this purpose, we exploited a negative photochromic T-type merocyanine that engages in a covalent exchange with an aniline nucleophile to provide an imine. Since the merocyanine can quantitatively be converted into its spiropyran isomer that is nonreactive in the exchange, the system can be shifted and trapped in the static spiropyran state. In the dark, however, the system thermally reverts back to the dynamic merocyanine that re-engages in the exchange. The process of shifting/trapping and re-equilibration can be repeated multiple times. The system provides opportunities for designing materials that allow for spatial and temporal control over their dynamic properties.

## Graphical abstract

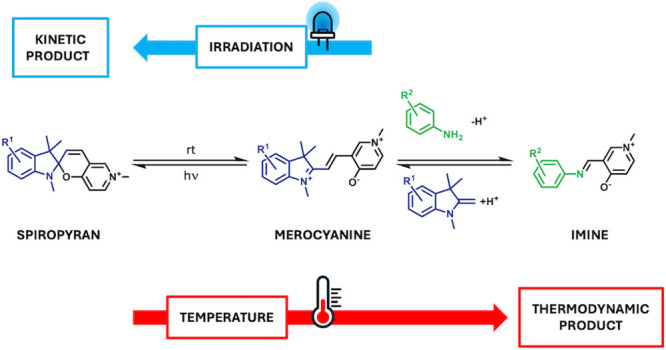

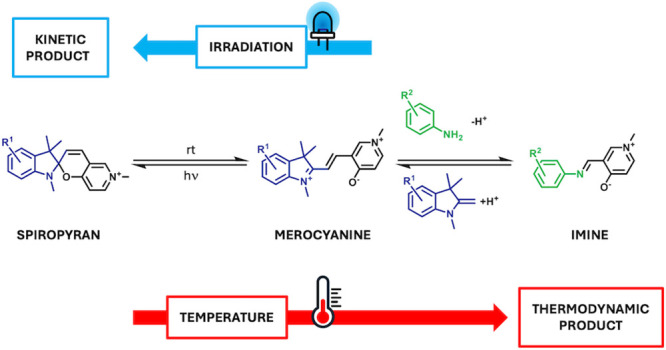

## Introduction

Dynamic covalent chemistry revolves around the reversible formation and cleavage of covalent bonds and, as a result, allows for the dynamic exchange of specific chemical constituents. The equilibrium composition of such systems is thermodynamically controlled, favoring formation of the most stable product(s).^1,2^ The stability of the individual components strongly depends on the environment of the system and can be altered by multiple stimuli such as pH,^3^ the presence of certain ions,^4^ and light.^5–9^ The latter, however, offers the major advantage that it can be applied in a noninvasive fashion with high spatial and temporal resolution. Thus, illumination allows the system to be reversibly changed without the addition of another component. Key to obtaining a dynamic covalent system that can be modulated and even driven by light is the integration of at least one photochromic component, i.e. photoswitch.^2^

Initially, we exploited diarylethenes (DAEs) to control dynamic bond formation (Figure 1). In our first approach, the reversible Diels–Alder reaction between furan and maleimide was controlled by creating a furan-containing DAE (**1o**), where depending on the state of the switch, the Diels–Alder reaction was either inhibited or activated by light (Figure 1a).^6^ In this system, only open DAE **1o** can undergo the desired Diels–Alder reaction. Photocyclization of **1o** to its closed isomer **1c** removes the reactive diene moiety and prevents reaction with the maleimide, thus locking the starting composition of the system. In addition, the Diels–Alder adduct **2o** can undergo electrocyclic ring closure as well, in which the double bond from the oxanorbornene skeleton is repositioned and prevents the retro-Diels–Alder reaction. Despite its superior optical control, the overall system suffers from the high temperatures needed to induce the retro-Diels–Alder reaction and reobtain DAE **1o**.

**Figure 1:**
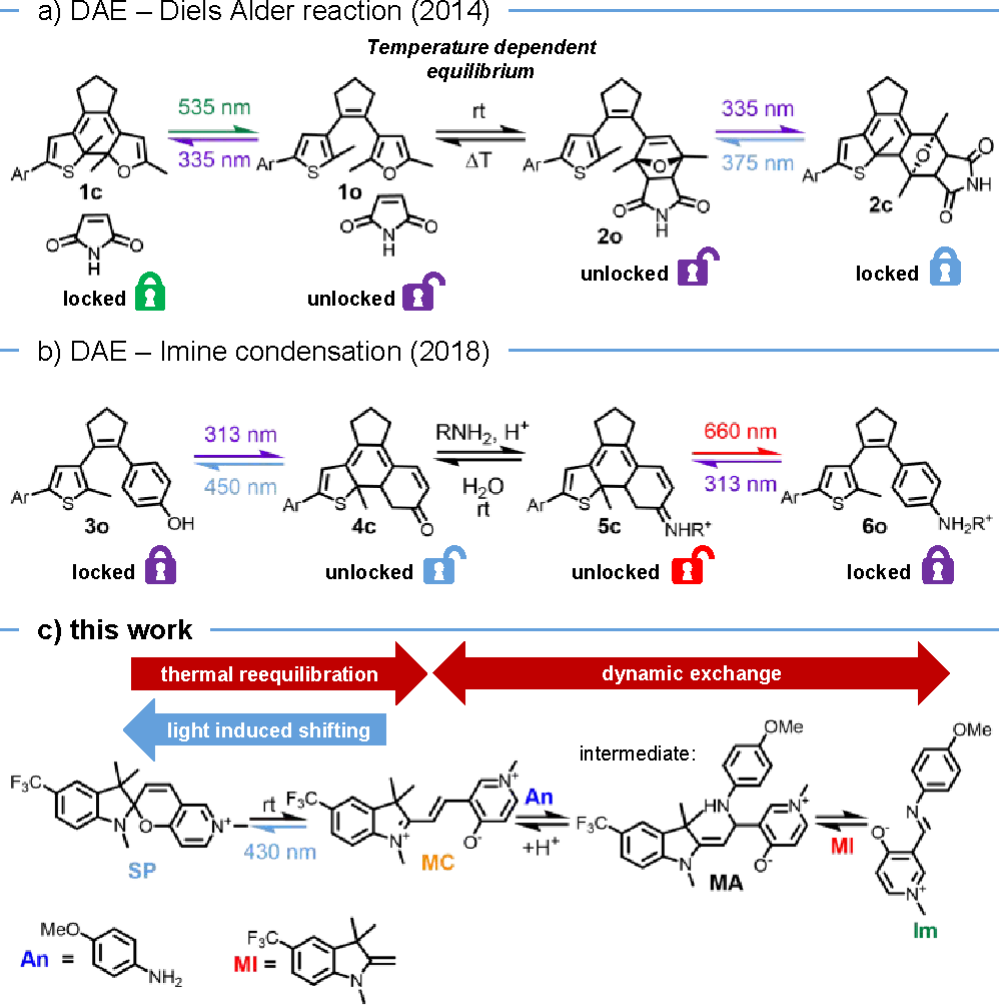
Light-driven dynamic covalent exchange systems: DAEs to drive (a) Diels–Alder reactions^6^ and (b) imine condensation and hydrolysis^7^ as well as (c) spiropyrans driving merocyanine-imine exchange (this work).

As a result, we subsequently focused on dynamic covalent reactions occurring at ambient conditions, in particular, imine exchange (Figure 1b).^7^ Our approach made use of phenol DAE **3o** as a light-activatable electrophile. Photocyclization breaks the aromaticity of the phenol moiety, and subsequently, the formed enol intermediate tautomerizes to the more stable ketone **4c**. At room temperature and in the presence of acid, the generated carbonyl electrophile **4c** reacts with N-based nucleophiles, such as primary amines, to yield iminium ions **5c**. The latter exhibits a dramatically red-shifted absorption band in the visible spectrum, which enables selective irradiation and allows the system to be shifted and locked either at the condensation or hydrolysis products, i.e., **6o** and **3o**, respectively.

Both DAE systems, however, require irradiation with UV light to either activate or inhibit dynamic bond formation. In material applications, this is typically problematic due to the low penetration depth of UV light, in addition to radiation damage that potentially limits cyclability. Although we have been able to shift DAE ring closure to the visible region,^10^ we sought a more general way to shift the equilibrium solely with visible light. Recently, we disclosed that spiropyrans are able to undergo dynamic covalent reactions enabling the exchange of the indoline moiety in the reactive merocyanine form, leading to the formation of a new spiropyran derivative.^11^ Although we were able to gain detailed mechanistic insight into the purely thermal exchange, the initial system did not allow us to control and drive its composition photochemically—until now.

Using 6-pyridinium-fused merocyanines that are more stable than their corresponding spiropyran isomers in polar solvents^12^ allowed us to exploit negative T-type photochromism and use visible light to quantitatively convert the electrophilic merocyanine into the inactive spiropyran form. Instead of using methylene indolines as nucleophiles in exchange reactions,^11^ we investigated amines, which are extremely versatile building blocks^7,8,13^ and known to react with merocyanines.^14^ Herein, we describe the resulting merocyanine-imine exchange system (Figure 1c), which allows for selective excitation and photochemical conversion of the reactive merocyanine to the nonreactive spiropyran using blue light. Due to its metastability, the spiropyran undergoes thermal back-switching to re-engage in the exchange equilibrium, and thereby, the composition of the dynamic covalent system can be shifted controllably with one wavelength only. The overall light-driven exchange process is reversible and can be repeated multiple times. Importantly, this new exchange system avoids the use of UV light, and shifting the equilibrium with visible light is much faster when compared to our prior DAE imine exchange system. In addition, thermal re-equilibration takes advantage of relatively mild temperatures to avoid fatigue and enhance the robustness of the system.

## Results and Discussion

The first successful merocyanine-imine exchange was observed with trifluoromethylated pyridinium-fused merocyanine **MC** and *p*-anisidine **An** by in situ ^1^H NMR experiments (Figure 2). Both components were dissolved in degassed methanol-d_4_ in equimolar amounts (15 mM), and the solution was transferred into an NMR tube. The tube was sealed under an argon atmosphere, and an initial ^1^H NMR spectrum was recorded (see Figure S2a in the Supporting Information). Already during sample preparation, taking approximately 10 min, the exchange nearly reached thermodynamic equilibrium; within 1 h, the system was fully equilibrated. The formation of the corresponding imine (**Im**) could be clearly observed through the distinct imine proton (H_e_) at 8.93 ppm as well as through the aromatic signals of H**_d_** at 8.56 ppm and H**_b_** at 7.84 ppm. At thermodynamic equilibrium, a distribution of **SP**: **MC**: **MA**: **Im** = 4:38:27:31 was reached.

**Figure 2:**
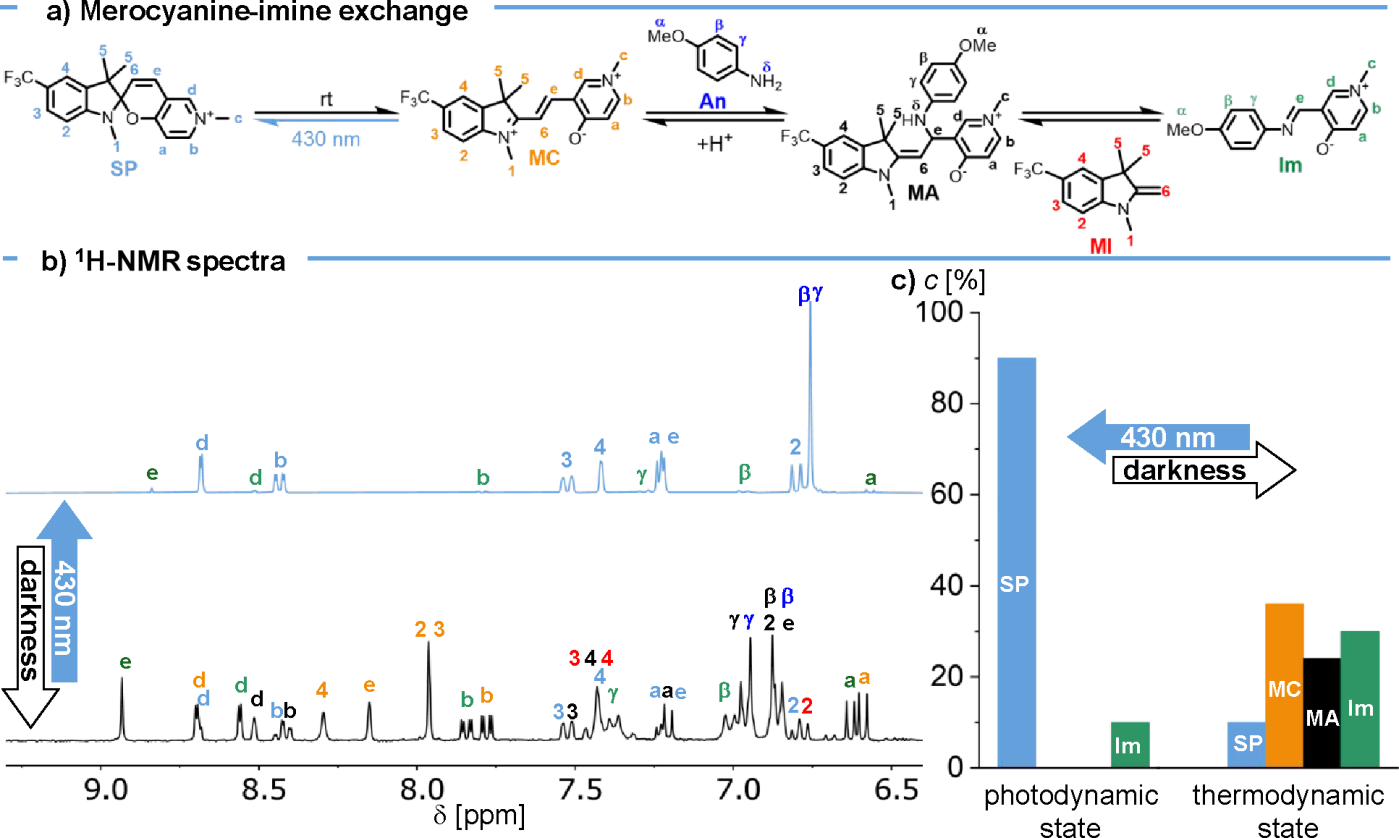
(a) Merocyanine-imine exchange via the formation of **MA**. Equilibrium shift through illumination with 430 nm due to formation of the closed, inactive isomer. (b) In situ ^1^H NMR monitoring of the merocyanine-imine exchange in cycle 1 (initial: *c* (**MC**) = 15 mM, *c* (**An**) = 15 mM in in methanol-*d*_4_); top spectrum was measured after reaching photodynamic equilibrium state and bottom spectrum after thermal re-equilibration. (c) Resulting **MC**, **Im**, **MA,** and **SP** concentration at the photodynamic and thermodynamic state determined by integrating the H_b_ signals at 8.44 ppm (**SP**), 8.40 (**MA**), 7.84 (**Im**), and 7.75 (**MC**) ppm.

Comparing the absorption spectra of both exchanging species, i.e., **MC** and **Im**, reveals a significant spectral separation (Δλ_max_ = 84 nm) between their absorption maxima (Figure 3), enabling the selective excitation of **MC**. Moreover, photochemical conversion is highly efficient and requires only short irradiation times of ca. 5 min with 430 nm for quantitative **SP** formation, even at NMR concentrations (see Figures S6 and S7 in the Supporting Information). This selective and quantitative **MC → SP** photoconversion enables us to control the dynamic covalent exchange system by trapping the reactive **MC** species as its nonreactive **SP** isomer.

**Figure 3:**
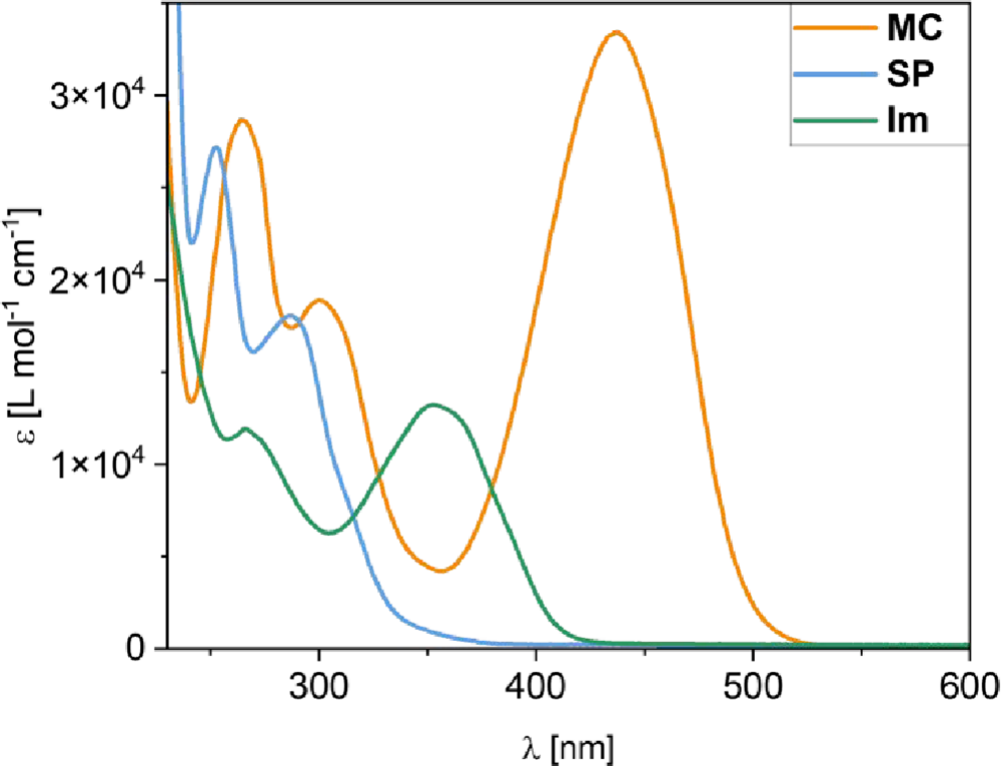
Comparison of the absorbance spectra of **MC**, **SP**, and **Im** in methanol at 20 °C.

In a proof-of-concept shifting experiment, the initially formed thermodynamic equilibrium state composed of **SP**: **MC**: **MA**: **Im** = 4:38:27:31 (see above) was irradiated with 430 nm for 5 h, resulting in the formation of a new photodynamic equilibrium state, characterized by the complete disappearance of **MC** and **MA** and pronounced enrichment of **SP** (Figure 2b). Upon such 430 nm irradiation, a ratio of **SP**: **Im** = 9:1 is reached in photodynamic equilibrium, and no fatigue was observed. Upon storing the corresponding sample in the dark for 40 h, thermodynamic equilibrium was re-established, resulting in a distribution of **SP**: **MC**: **MA**: **Im** = 10:36:24:30, closely resembling the initially obtained thermal equilibrium composition (Figure 2b).

To gain more insight into this exchange system, the very same sample was irradiated once again for 30 min with 430 nm until the photodynamic equilibrium was reached; afterward, the thermal relaxation was monitored over 10 h by in situ ^1^H NMR. One cycle was completed after measuring an additional ^1^H NMR spectrum after a 30-h period. This full cycle procedure was repeated two times (Figures S3 and S4 in the Supporting Information).

The light-driven shifting experiment was repeated multiple times and showed similar distributions in each case. The **SP** amount at photodynamic equilibrium and the **MC** amount at thermodynamic equilibrium increased slightly over the cycles, which could be explained by a small amount of **An** fatigue due to residual oxygen in the system.

In addition, we exploited the effect of using an excess of nucleophilic exchange partner in the system and carried out the same cycle experiment with 3 equiv of **An** (see Figure S5 in the Supporting Information). Due to the larger amount of **An**, the thermodynamic equilibrium shifted to a distribution of **SP**: **MC**: **MA**: **Im** = 8:14:30:48, whereas at photodynamic equilibrium, **SP**: **MC**: **MA**: **Im** = 69:0:9:22 was reached. In addition, the ring opening of **SP** in the dark was monitored over 100 h (see Figure S7 in the Supporting Information) to compare the kinetics of thermal **SP** decay with the exchange experiment. The outcome nicely corroborates the finding of Shiraishi et al. that nucleophiles such as thiolates indirectly affect the thermal half-life of 6-nitrospiropyran via a reversible attack on the intermediately formed corresponding merocyanine.^15^

Furthermore, we exploited a stronger aniline nucleophile to investigate the distribution of the different species in both equilibrium states. Using *N*,*N*-dimethyl-1,4-phenylenediamine (**An2**, 1 equiv) resulted in the formation of more of the imine species (**Im2**) in the thermodynamic equilibrium state (**SP**:**MC**:**MA2**:**Im2** = 3:17:27:52). However, use of the stronger nucleophile limited the ability to shift the system with blue light, resulting in a ratio of **SP**:**MC**:**MA2**:**Im2** = 59:5:5:31 after 150 min with 430 nm irradiation due to more rapid thermal relaxation competing with the light-driven shifting (see Figure S6 in the Supporting Information).

From the results above, we conceptually envision our merocyanine-imine exchange system to be composed of rapidly interconverting **MC** and **Im** species mechanistically connected via intermediate Michael adduct **MA** that enables fast and reversible exchange via low barriers (Figure 4). Blue light irradiation quantitatively removes the reactive **MC** species and traps it as a nonreactive spiropyran isomer. Upon removal of the light source, the **MC** isomer is thermally regenerated yet only at a slow rate due to a relatively high barrier and subsequently re-engages in rapid thermal exchange with the present nucleophile. Therefore, light serves to create a temporary state of low or no reactivity that locks dynamic exchange in the system, which can be recovered in the dark. Conceptually similar dissipative systems have been constructed^16^ and used to control the assembly of azobenzene-covered nanoparticles.^17,18^

**Figure 4:**
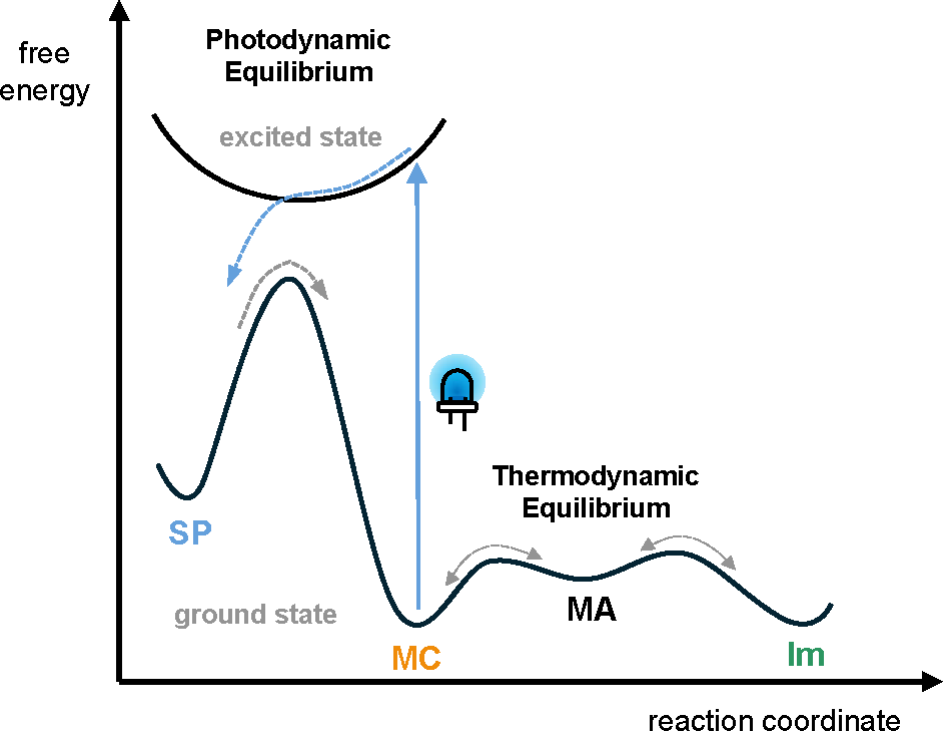
Hypothetical energy profile illustrating the operating mechanism of shifting the merocyanine-imine exchange with light.

## Conclusion

In the described work, we demonstrated a new light-driven merocyanine-imine exchange system, in which the thermodynamic equilibrium can be controlled and shifted by visible light. We have been able to observe the key intermediate enabling the exchange, identify each participating species, and fully characterize the mechanism operating in the exchange system.

The system allows external modulation of the covalent connection of enamine and aniline nucleophiles to yield spiropyran and imine adducts, respectively. The process does not require the use of UV light and instead uses visible light of one wavelength (430 nm) only and can be repeated multiple times. For these reasons, we foresee the integration of our system into various types of materials to remote control their properties, and, in particular, static and dynamic states by light.

## Supplementary Material



## Notes

Materials and methods, experimental procedures, including synthesis and compound characterization, merocyanine-imine exchange, and photochemical and thermal isomerization behavior, as well as spectral data (PDF)
