# Supplementary material for: Shifting Merocyanine-Imine Exchange with Visible Light
Source: J Am Chem Soc. 2026 Feb 5;148(6):6188–92. doi: 10.1021/jacs.5c17606 (PMC12921852; doi:10.1021/jacs.5c17606)
Supplement: Supplementary file 1 [file ja5c17606_si_001.pdf]

Supporting Information

Shifting Merocyanine-Imine Exchange with Visible Light

Alwin Drichel[†,‡] and Stefan Hecht[†,‡,§,*]

† DWI-Leibniz Institute for Interactive Materials, 52074 Aachen, Germany
‡ Institute of Technical and Macromolecular Chemistry, RWTH Aachen University, 52074 Aachen, Germany
§ Department of Chemistry & Center for the Science of Materials Berlin, Humboldt-Universität zu Berlin, 12489 Berlin, Germany

Email: sh@hu-berlin.de

Table of Contents

1. Materials and Methods 1
2. Procedures 2
  2.1 Synthesis and Compound Characterization 2
  2.2 Merocyanine-Imine Exchange I 8
  2.3 Merocyanine-Imine Exchange II 14
  2.4 Photochemical ring-closure and thermal ring-opening 16
3. Appendix 19
  3.1 NMR Spectra 19
4. Reference 29

1. Materials and Methods

General remarks: Chemicals and dry solvents were purchased by TCI Deutschland GMBH or Th. Geyer and were used without further work up. Technical grade solvents were distilled prior to their use. TLC plates were coated with SiO_2-60 UV254 and were obtained from Merck. Column chromatography was performed with silica gel 60 (particle size 40 – 63 mm from VWR)

NMR Spectra were recorded on a Bruker AV III 400MHz spectrometer (400 MHz for ^{1}H, 100 MHz for ^{13}C and 376 MHz for ^{19}F) or on a Bruker AV III 300MHz spectrometer (300 MHz for ^{1}H, 75 MHz for ^{13}C and 282 MHz for ^{19}F) at room temperature. The abbreviation for multiplicities is as follows: singlet (s), doublet (d), triplet (t), quartet (q) and multiplet (m). Coupling constants (J) are given in Hz and chemical shifts (d) are given in ppm. NMR spectra are provided in the appendix.

In situ ^{1}H-NMR experiments were conducted *via* Bruker AV III 300MHz spectrometer. The prepared samples were put immediately in the NMR instrument for the NMR study. Irradiation of the NMR samples was performed with a Thorlab M430L4 mounted LED (500 mW) with LTN330-A collimation and focusing tube.

UV/vis spectroscopy was performed on Agilent Cary 60 instruments in 3 mL volume quartz cuvettes (l = 1.00 cm). For UV/vis spectra, an Oriel 500W Hg(Xe)-short-arc lamp model LSB740 in an Oriel arc lamp box LSH302 was used. It was equipped with an Oriel 300 mm grating monochromator model MSH-300, a shutter, and a light guide by Oriel.

The concentration of the merocyanine (**MC**), spiropyran (**SP**), Michael adduct (**MA**) as well as of the imine (**Im**) were determined through the H_b signals in the ^{1}H-NMR (8.44 ppm (**SP**), 8.40 (**MA**), 7.81 (**Im**) and 7.75 ppm (**MC**)). The concentration of merocyanine at time *t* (c_t(**MC**)) is calculated as shown in equation 1:

$$c_t(\mathbf{MC}) = \frac{a_\mathrm{I}(\mathbf{MC})}{a_\mathrm{I}(\mathbf{MC}) + a_\mathrm{I}(\mathbf{SP}) + a_\mathrm{I}(\mathbf{MA}) + a_\mathrm{I}(\mathbf{Im})} \cdot c_0(\mathbf{MC}) \qquad [1]$$

With a_I describing the integration area of the distinct H_b signals in the ^{1}H-NMR spectra.

2. Procedures

2.1 Synthesis and Compound Characterization

3-Formyl-4-hydroxy-1-methylpyridin-1-ium iodide (**1**)

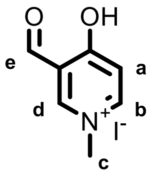

1

A vial was charged with 4-hydroxy-3-pyridinecarboxaldehyde (0.616 g, 1 equiv., 5 mmol), iodomethane (0.62 mL, 2 equiv., 10 mmol) and acetonitrile (10 mL, 0.5 M). The suspension was stirred at 80 °C for 24 h. The mixture was let to cool to rt and the precipitate was collected by filtration. The filtered precipitate was washed with a small amount of acetonitrile and diethyl ether to obtain 0.652 mg (2.46 mmol, 49 %) of the compound as light yellow solid.

^{1}H-NMR (400 MHz, DMSO-d$_6$): δ [ppm] = 10.15 (s, 1H, **e**), 8.71 (s, 1H, **d**), 8.28 (d, J = 7.4, 1H, **b**), 7.00 (d, J = 7.4 Hz, 1H, **a**), 3.98 (s, 3H, **c**).

^{13}C-NMR (100 MHz, DMSO-d$_6$): δ [ppm] = 187.80, 172.96, 146.56, 146.54, 121.58, 118.49, 45.13.

1,2,3,3-Tetramethyl-5-(trifluoromethyl)-3H-indolium iodide (**2**)

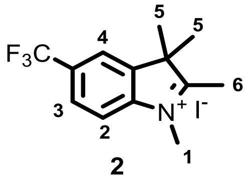

2

2 was synthesized according to the procedure described in literature[1] to obtain 1.55 g (4.2 mmol, 60%) of the compound as white solid.

^{1}H-NMR (400 MHz, MeCN-d$_3$): δ [ppm] = 8.09 (s, 1H, **4**), 7.97 (d, J = 8.3 Hz, 1H, **3**), 7.91 (d, J = 8.5 Hz, 1H, **2**), 3.98 (s, 3H, **1**), 2.78 (s, 3H, **6**), 1.60 (s, 6H, **5**).

^{19}F-NMR (376 MHz, MeCN-d$_3$): δ [ppm] = -62.59

HR-MS (ESI+): m/z calculated for [$C_{13}H_{14}F_3N+H^+$] **242.117517**, found **242.115111**.

1-Methyl-3-(2-(1,3,3-trimethyl-5-(trifluoromethyl)-3H-indol-1-ium-2-yl)vinyl)pyridin-1-ium-4-olate iodide (**MC**)

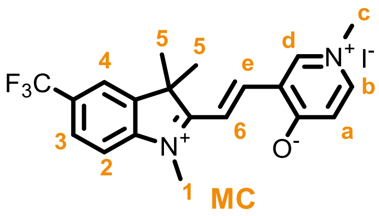

A beaker was charged with 1,2,3,3-tetramethyl-5-(trifluoromethyl)-3H-indolium iodide **2** (443 mg, 1.2 equiv. 1.2 mmol) and a 1M NaOH solution (0.25 M, 4.8 mL). The mixture was stirred for 30 min at rt and was extracted with dichloromethane. The combined organic phase was dried with $MgSO_4$. The organic phase was removed under reduced pressure and the residue oil was redissolved in acetonitrile (0.6 M, 2 mL). 4-hydroxy-3-pyridinecarboxaldehyde (123 mg, 1 equiv., 1 mmol) and iodomethane (0.125 mL, 2 equiv., 2 mmol) were added to the solution and the suspension was refluxed for 24h. After the suspension was let to cool to rt, the precipitate was collected by filtration, washed with a small amount of acetonitrile and diethyl ether. The solid was dissolved in methanol and reprecipitated in diethyl ether to obtain 350 mg (0.72 mmol, 72 %) of the compound as red solid.

^{1}H-NMR (400 MHz, DMSO-d$_6$): δ [ppm] = 8.81 (s, 1H, **d**), 8.62 (d, J = 15.6 Hz, 1H, **6**), 8.36 (s, 1H, **4**), 8.26 (d, J = 15.6 Hz, 1H, **e**), 8.07 (d, J = 8.5 Hz, 1H, **2**), 7.99 (d, J = 8.6 Hz, 1H, **3**), 7.78 (d, J = 7.4 Hz, 1H, **b**), 6.43 (d, J = 7.4 Hz, 1H, **a**), 3.96 (s, 3H, **1**), 3.81 (s, 3H, **c**), 1.82 (s, 6H, **5**).

^{13}C-NMR (100 MHz, DMSO-d$_6$): δ [ppm] = 184.75, 176.45, 151.80, 151.44, 144.88, 144.08, 141.27, 128.76, 128.44, 126.47, 125.39, 122.68, 120.81, 120.62, 120.30, 115.50, 111.21, 51.95, 43.93, 34.11, 25.52.

^{19}F-NMR (376 MHz, DMSO-d$_6$): δ [ppm] = -60.09

^{1}H-NMR (300 MHz, MeOD-d$_4$): δ [ppm] = 8.70 (d, J = 2.3 Hz, 1H, **d**), 8.30 (s, 1H, **4**), 8.15 (s, 1H, **e**), 8.00 – 7.92 (m, 2H, **2**, **3**), 7.78 (dd, J = 7.6, 2.3 Hz, 1H, **b**), 6.59 (d, J = 7.6 Hz, 1H, **a**), 4.10 (s, 3H, **1**), 3.91 (s, 3H, **c**), 1.89 (s, 6H, **5**).

HR-MS (ESI+): m/z calculated for [$C_{20}H_{20}F_3N_2O^+$] **361.152224** found **361.153448.**

1,3,3,6'-Tetramethyl-5-(trifluoromethyl)spiro[indoline-2,2'-pyrano[3,2-c]pyridin]-6'-ium iodide (**SP**)

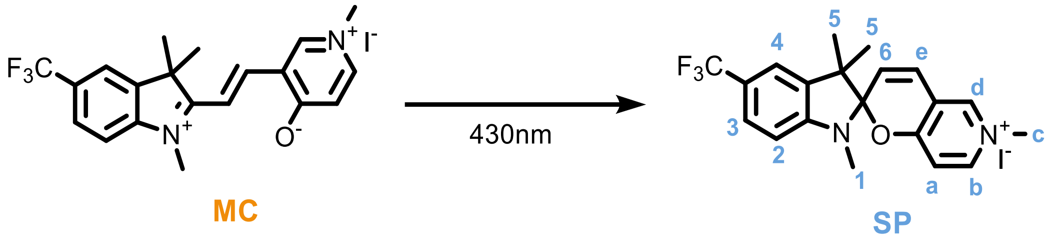

An NMR tube was charged with 1-methyl-3-(2-(1,3,3-trimethyl-5-(trifluoromethyl)-3H-indol-1-ium-2-yl)vinyl)pyridin-1-ium-4-olate iodide (**MC**) and dissolved in either DMSO-d_6 or CD_3OD. The tube was irradiated with 430 nm for 5 min or 15 min to fully convert **MC** to **SP**.

^{1}H-NMR (400 MHz, DMSO-d_6): δ [ppm] = 8.88 (d, J = 1.8 Hz, 1H, **d**), 8.59 (dd, J = 7.0, 1.8 Hz, 1H, **b**), 7.59 – 7.52 (m, 2H, **3**, **4**), 7.43 (d, J = 7.0 Hz, 1H, **a**), 7.27 (d, J = 10.6 Hz, 1H, **e**), 6.88 (d, J = 8.6 Hz, 1H, **2**), 6.30 (d, J = 10.6 Hz, 1H, **6**), 4.12 (s, 3H, **c**), 2.81 (s, 3H, **1**) 1.29 (s, 3H, **5**), 1.18 (s, 3H, **5**).

^{13}C-NMR (100 MHz, DMSO-d_6): δ [ppm] = 164.88, 150.40, 147.06, 143.96, 136.72, 126.81, 126.57, 125.31, 123.97, 121.04, 120.72, 119.52, 117.77, 113.80, 109.98, 107.62, 52.94, 46.83, 29.02, 25.75, 19.56.

^{19}F-NMR (376 MHz, DMSO-d_6): δ [ppm] = -58.96

^{1}H-NMR (300 MHz, MeOD-d_4): δ [ppm] = 8.68 (d, J = 1.8 Hz, 1H, **d**), 8.44 (dd, J = 7.1, 1.9 Hz, 1H, **b**), 7.57 – 7.48 (m, 1H, **3**), 7.42 (d, J = 1.4 Hz, 1H, **4**), 7.27 – 7.19 (m, 2H, **a**, **e**), 6.80 (d, J = 8.2 Hz, 1H, **2**), 4.19 (s, 3H, **c**), 2.88 (s, 3H, **1**), 1.36 (s, 3H, **5**), 1.26 (s, 3H, **5**).

3-(((4-Methoxyphenyl)imino)methyl)-1-methylpyridin-1-ium-4-olate iodide (**Im-I**)

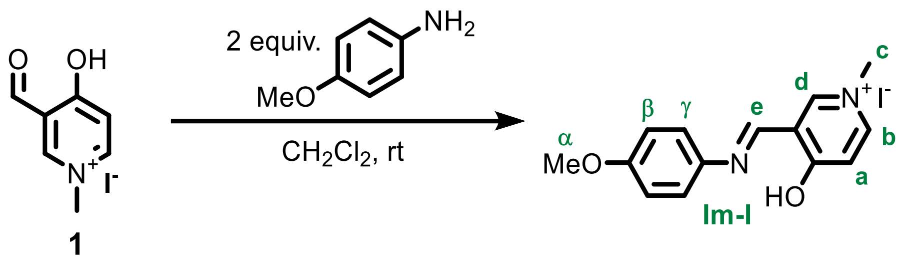

A vial was charged with *p*-anisidine (**An**) (123 mg, 2 equiv., 1 mmol), 3-formyl-4-hydroxy-1-methylpyridin-1-ium iodide (**1**) (133 mg, 1 equiv., 0.5 mmol) and dichloromethane (2 mL, 0.5 M). The suspension was stirred for 24 h and the precipitate was collected by filtration. The filtered precipitate was recrystallized in ethanol and water to obtain 139 mg (0.374 mmol, 75%) of the compound as orange crystalline solid.

^{1}H-NMR (400 MHz, DMSO-d$_6$): *δ* [ppm] = 9.33 (s, 1H, **e**), 8.77 (d, *J* = 2.2 Hz, 1H, **d**), 8.12 (dd, *J* = 7.7, 2.1 Hz, 1H, **b**), 7.72 (d, *J* = 9.1 Hz, 2H, γ), 7.16 (d, *J* = 9.0 Hz, 2H, β), 6.85 (d, *J* = 7.6 Hz, 1H, **a**), 3.97 (s, 3H, **c**), 3.84 (s, 3H, α).

^{13}C-NMR (100 MHz, DMSO-d$_6$): *δ* [ppm] = 176.92, 160.29, 159.12, 152.24, 144.05, 130.68, 122.64, 120.14, 115.22, 114.31, 55.78, 45.07.

^{1}H-NMR (300 MHz, CD$_3$OD): *δ* [ppm] = 9.25 (s, 1H, **e**), 8.72 (d, *J* = 2.2 Hz, 1H, **d**), 8.01 (dd, *J* = 7.7, 2.2 Hz, 1H, **b**), 7.75 – 7.64 (m, 2H, γ), 7.21 – 7.02 (m, 2H, β), 6.84 (d, *J* = 7.6 Hz, 1H, **a**), 4.01 (s, 3H, **c**), 3.89 (s, 3H, α).

HR-MS (ESI+): m/z calculated for [$C_{14}H_{15}N_2O_2^+$] **243.112804**, found **243.111184.**

3-(((4-Methoxyphenyl)imino)methyl)-1-methylpyridin-1-ium-4-olate (**Im**)

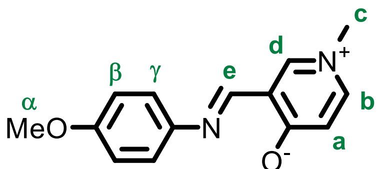

Im

Im was obtained by dissolving **Im-I** (2.78 mg, 1 equiv., 7.5 μmol) and triethyl amine (1 μL, 1 equiv., 7.5 μmol) in CD_3OD (0.5 mL, 15mM).

^{1}H-NMR (300 MHz, MeOD-d$_4$): δ [ppm] = 8.85 (s, 1H, **e**), 8.52 (d, J = 2.3 Hz, 1H, **d**), 7.80 (dd, J = 7.5, 2.3 Hz, 1H, **b**), 7.35 – 7.24 (m, 2H, γ), 7.03 – 6.92 (m, 2H, β), 6.57 (d, J = 7.5 Hz, 1H, **a**), 3.88 (s, 3H, **c**), 3.82 (s, 3H, α).3-(((4-

3-(((4-(dimethylamino)phenyl)imino)methyl)-4-hydroxy-1-methylpyridin-1-ium iodide (**Im-II**)

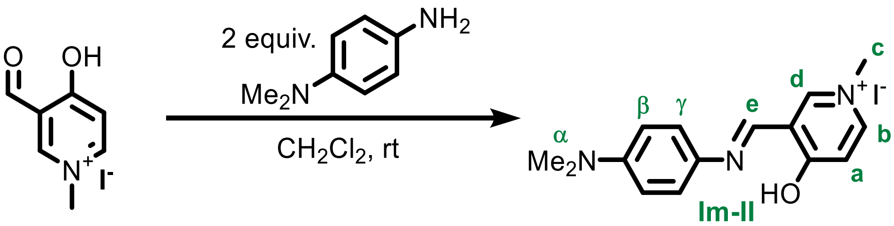

A vial was charged with N,N-Dimethyl-1,4-phenylendiamin (**An2**) (41 mg, 2 equiv., 0.3 mmol), 3-formyl-4-hydroxy-1-methylpyridin-1-ium iodide (**1**) (40 mg, 1 equiv., 0.15 mmol) and dichloromethane (0.3 mL, 0.5 M). The suspension was stirred for 24 h and the precipitate was collected by filtration to obtain 36 mg (0.09 mmol, 62%) of the compound as orange solid.

^{1}H-NMR (300 MHz, CD_3OD): δ [ppm] = 9.11 (s, 1H, **e**), 8.63 (d, J = 2.2 Hz, 1H, **d**), 7.97 (dd, J = 7.7, 2.2 Hz, 1H, **b**), 7.59 (d, J = 9.2 Hz, 2H, γ), 6.89 (d, J = 9.2 Hz, 2H, β), 6.79 (d, J = 7.7 Hz, 1H, **a**), 3.99 (s, 3H, **c**), 3.08 (s, 6H, α).

HR-MS (ESI+): m/z calculated for [$C_{14}H_{15}N_2O_2^+$] **256.144439**, found **256.145709.**

3-(((4-(dimethylamino)phenyl)imino)methyl)-1-methylpyridin-1-ium-4-olate (**Im2**)

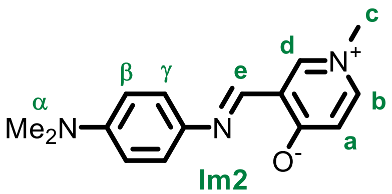

Im2

Im2 was obtained by dissolving **Im-II** (2.87 mg, 1 equiv., 7.5 μmol) and triethyl amine (1 μL, 1 equiv., 7.5 μmol) in CD_3OD (0.5 mL, 15mM).

^{1}H-NMR (300 MHz, MeOD-d$_4$): δ [ppm] = 8.99 (s, 1H, **e**), 8.56 (d, J = 2.2 Hz, 1H, **d**), 7.89 (dd, J = 7.5, 2.2 Hz, 1H, **b**), 7.46 (d, J = 9.1 Hz, 2H, γ), 6.85 (d, J = 9.1 Hz, 2H, β), 6.69 (d, J = 7.6 Hz, 1H, **a**), 3.94 (s, 3H, **c**), 3.04 (s, 6H, α).

2.2 Merocyanine-Imine Exchange I

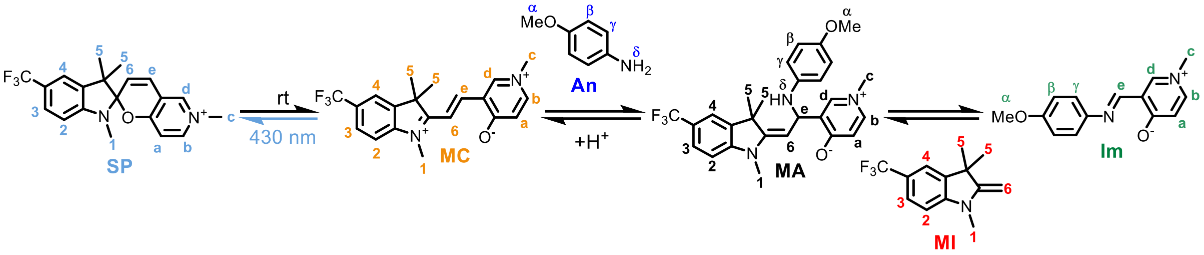

A vial was charged with 1-methyl-3-(2-(1,3,3-trimethyl-5-(trifluoromethyl)-3H-indol-1-ium-2-yl)vinyl)pyridin-1-ium-4-olate iodide (**MC**) (3.66 mg, 1 equiv., 7.5 mmol) and 1 or 3 equiv. of *p*-anisidine (**An**) (0.91 or 2.77 mg, 7.5 or 22.5 mmol). Argon-degassed deuterated methanol (0.5 mL, 15 mM) was added to the vial to fully dissolve the compounds and the reaction mixture was transferred into an NMR tube. The NMR tube was charged with argon for 1 min and was sealed. The exchange reaction was monitored *via* ^{1}H-NMR spectroscopy. After the thermodynamic equilibrium state was reached, the NMR tube was irradiated with 430 nm until the photodynamic equilibrium state was obtained. Afterwards the thermal re-equilibration was investigated, completing one cycle.

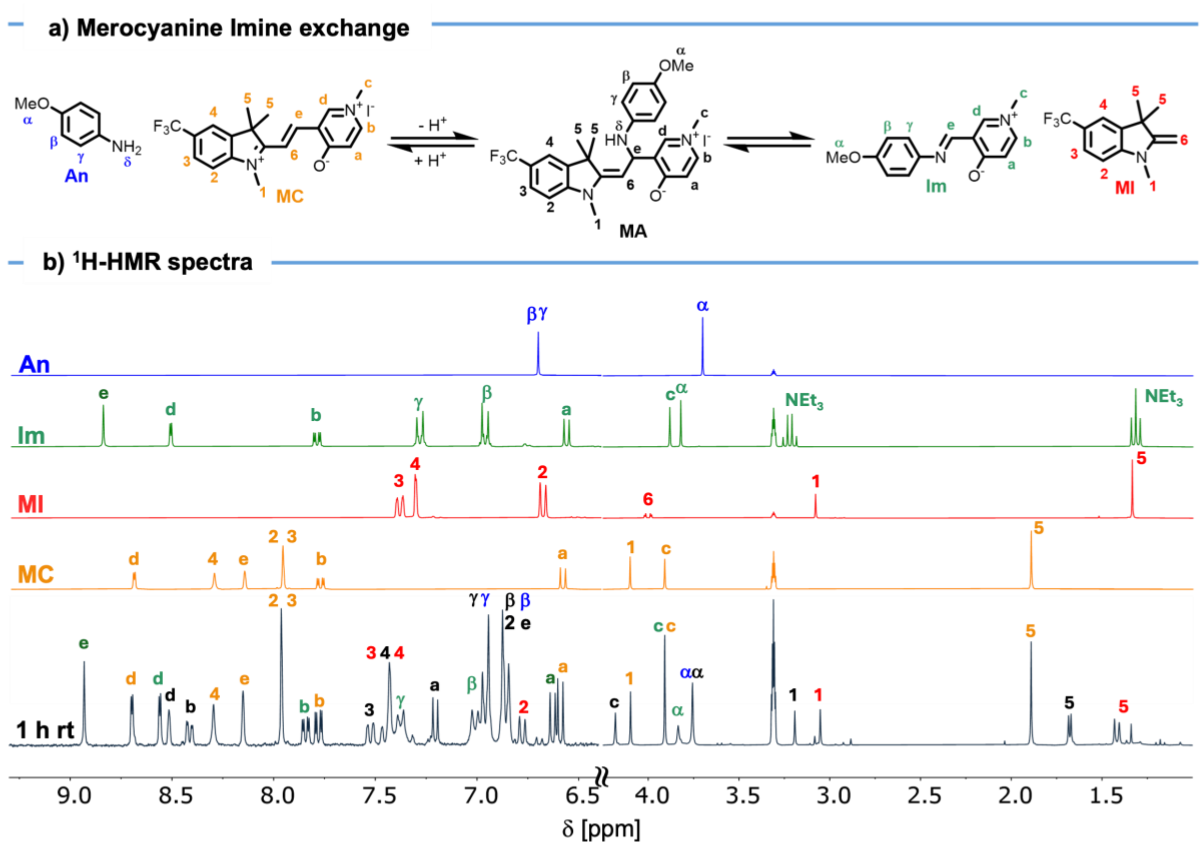

Figure S1. a) Merocyanine-imine exchange *via* the formation of **MA** b). Top four spectra: individual ^{1}H-NMR spectra of compounds present in the exchange in methanol-d$_4$, bottom ^{1}H-NMR spectrum of thermodynamic equilibrated merocyanine-imine exchange starting with **MC** and **An** (c (**MC**) = 15 mM, c (**An**) = 15 mM) in methanol-d$_4$.

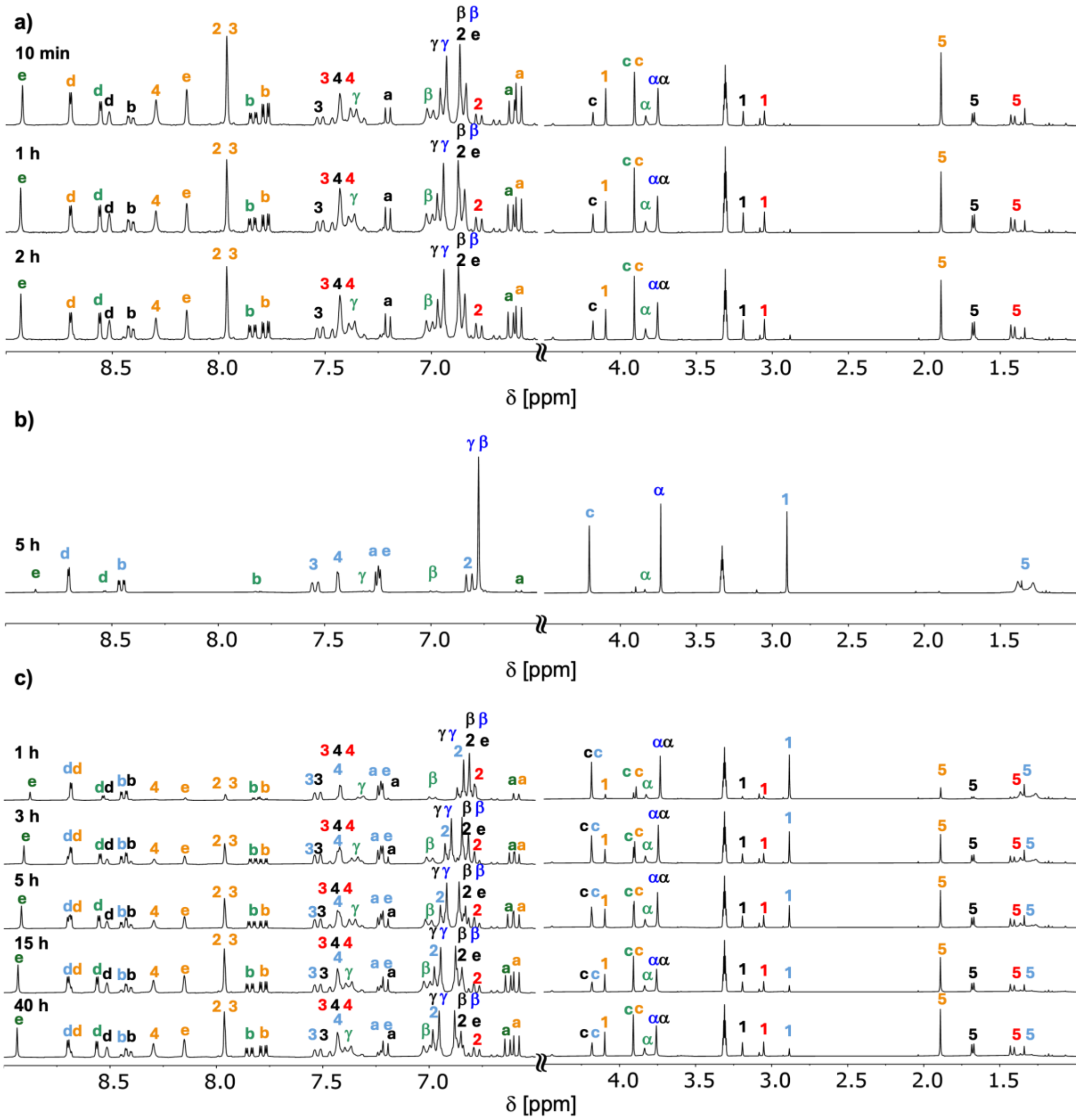

Figure S2. First merocyanine-imine exchange cycle: a) In situ ^{1}H-NMR monitoring of initial thermal equilibration starting with **MC** and **An** (c (**MC**) = c (**An**) = 15 mM in CD_3OD). b) ^{1}H-NMR spectrum after 5 h irradiation with 430 nm. c) In situ ^{1}H-NMR monitoring of thermal re-equilibration after the sample was irradiated with 430 nm.

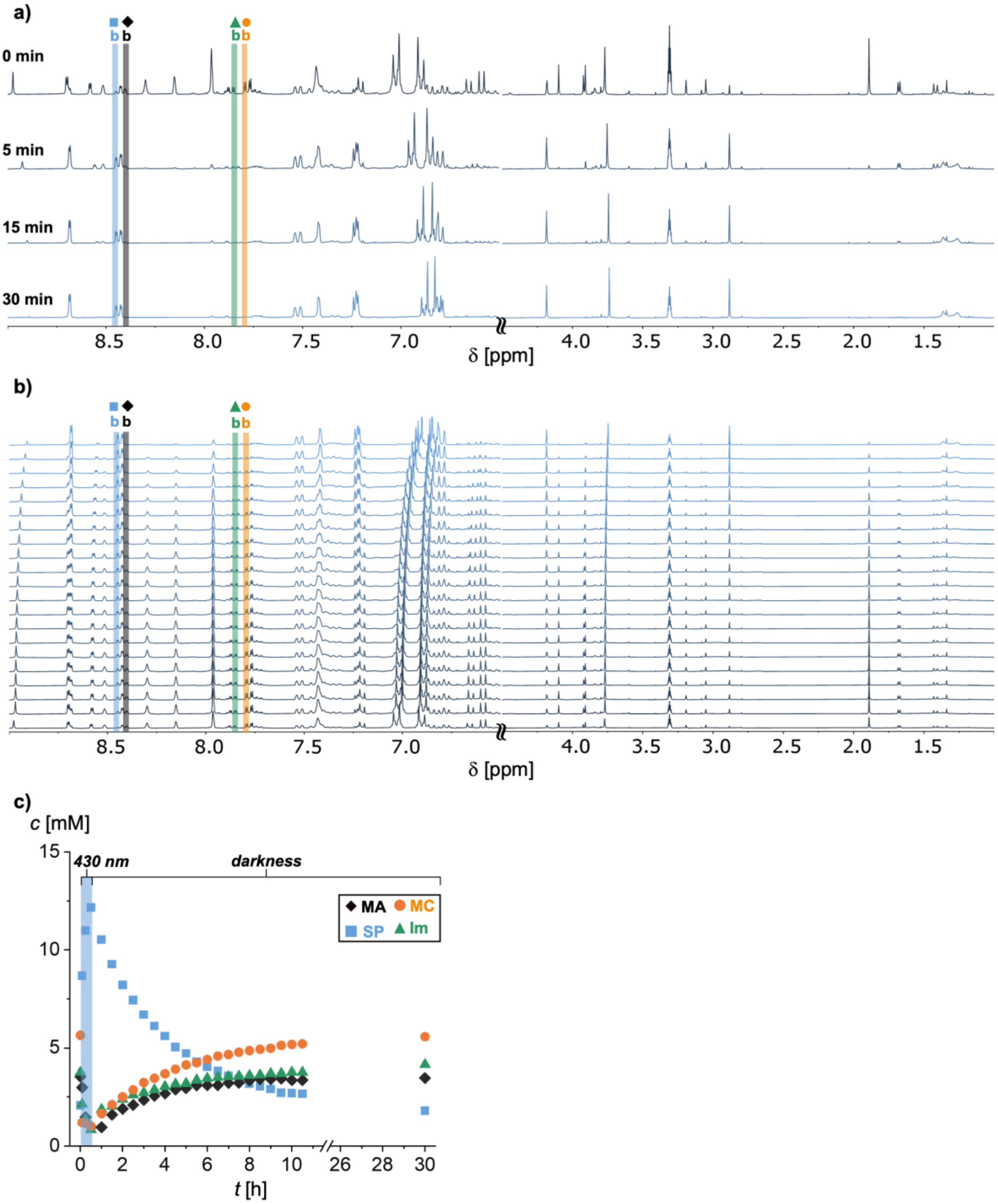

Figure S3. Second merocyanine-imine exchange cycle: a) In situ ^{1}H-NMR monitoring of photodynamic equilibration starting after thermal re-equilibration of the first cycle (irradiation with 430nm, Δt = 5, 15 and 30 min in CD_3OD). b) In-situ ^{1}H-NMR monitoring of thermal re-equilibration after the sample was irradiated with 430 nm (first 10 h Δt = 0.5 h afterwards measured at t = 30 h). c) Resulting **MC**, **Im**, **MA** and **SP** concentration evolution while irradiation and thermal re-equilibration determined by integration of the H_b signals at 8.44 ppm (**SP**), 8.40 (**MA**), 7.84 (**Im**) and 7.75 ppm (**MC**).

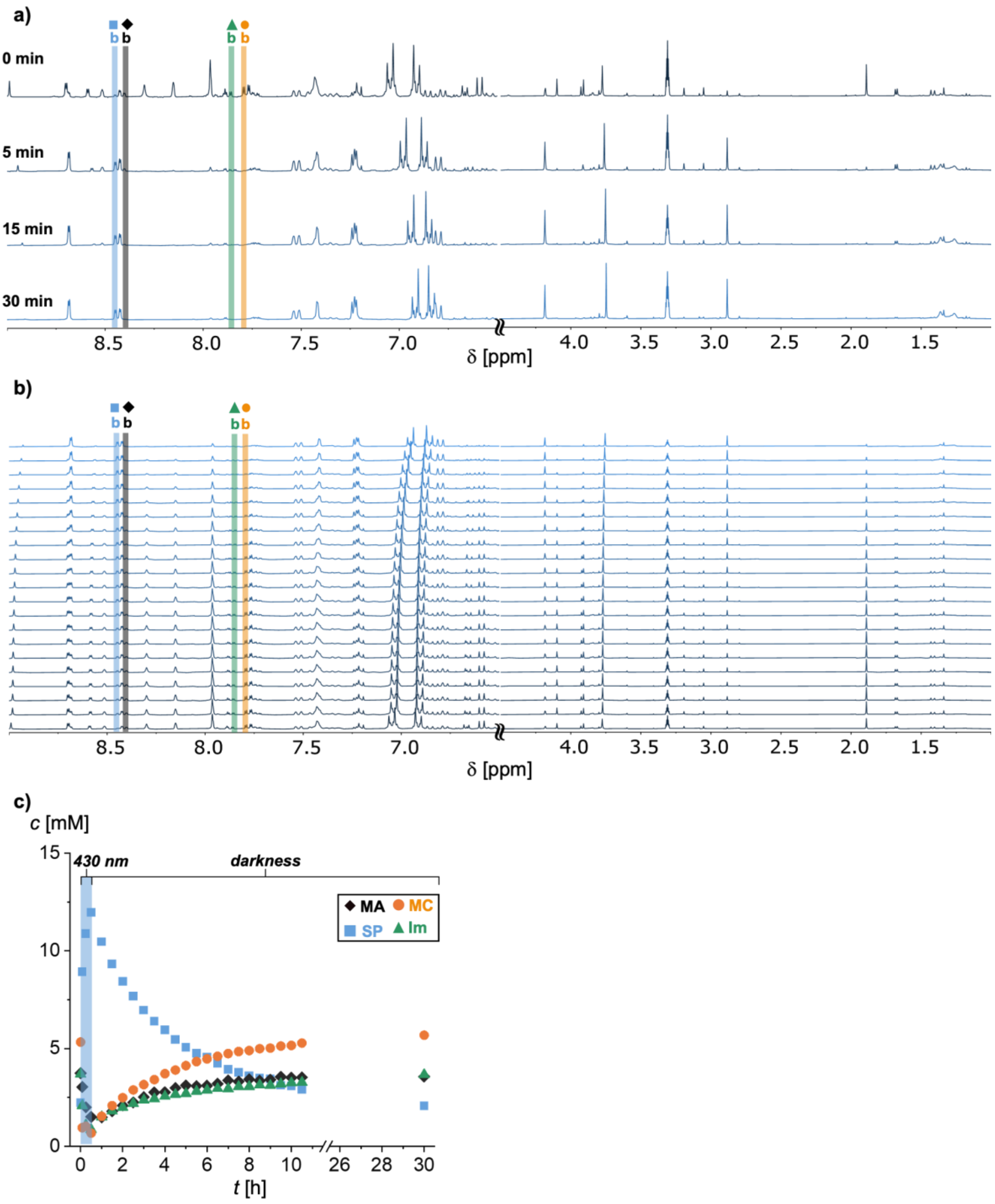

Figure S4. Third merocyanine-imine exchange cycle: a) In situ ^{1}H-NMR monitoring of photodynamic equilibration starting after thermal re-equilibration of the second cycle (irradiation with 430nm, Δt = 5, 15 and 30 min in CD_3OD). b) In situ ^{1}H-NMR monitoring of thermal re-equilibration after the sample was irradiated with 430 nm (first 10 h Δt = 0.5 h afterwards measured at t = 30 h). c) Resulting **MC**, **Im**, **MA** and **SP** concentration evolution while irradiation and thermal re-equilibration determined by integration of the H_b signals at 8.44 ppm (**SP**), 8.40 (**MA**), 7.81 (**Im**) and 7.75 ppm (**MC**).

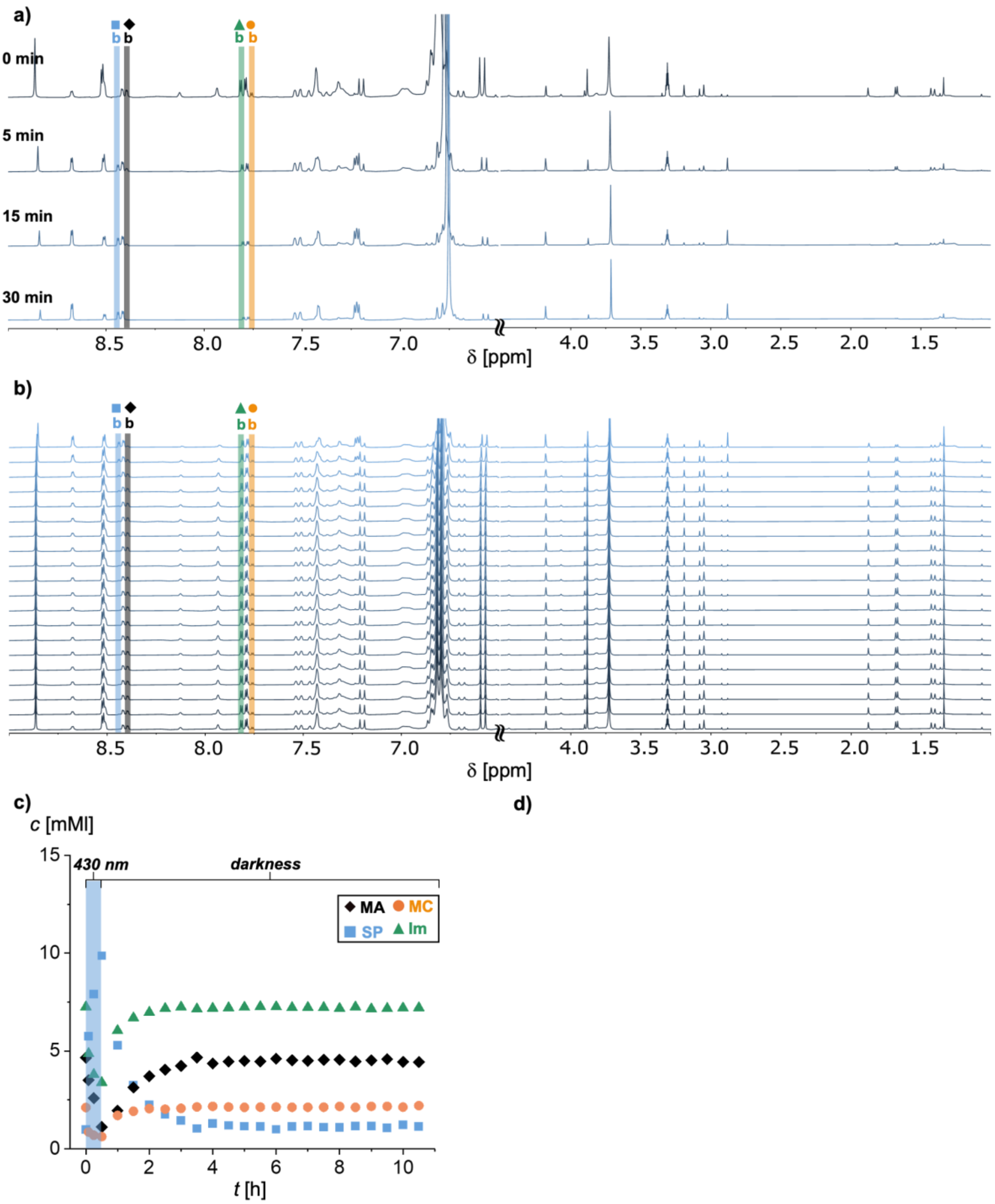

Figure S5. Merocyanine-imine exchange cycle using 3 equivalents of aniline **An** (*c* (**MC**) = 15 mM, *c* (**An**) = 45 mM in CD_3OD): a) In situ ^{1}H-NMR monitoring of photodynamic equilibration starting after thermal equilibration (irradiation with 430 nm, Δt = 5, 15, and 30 min in CD_3OD). b) In situ ^{1}H-NMR monitoring of thermal re-equilibration after the sample was irradiated with 430 nm (over 10 h Δt = 0.5 h). c) Resulting **MC**, **Im**, **MA**, and **SP** concentration evolution while irradiation and thermal re-equilibration determined by integration of the H_b signals at 8.44 ppm (**SP**), 8.40 (**MA**), 7.84 (**Im**) and 7.75 ppm (**MC**).

2.3 Merocyanine-Imine Exchange II

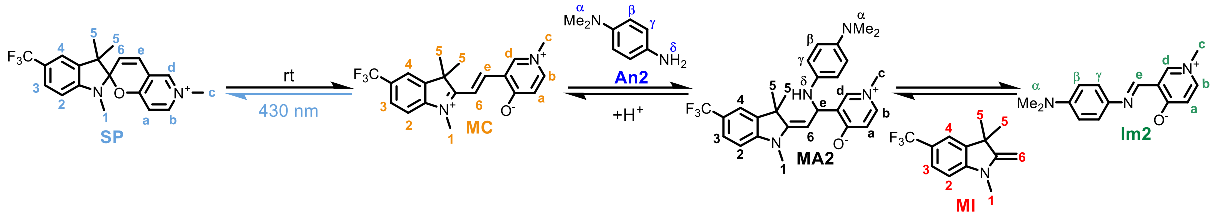

A vial was charged with 1-methyl-3-(2-(1,3,3-trimethyl-5-(trifluoromethyl)-3H-indol-1-ium-2-yl)vinyl)pyridin-1-ium-4-olate iodide (**MC**) (3.66 mg, 1 equiv., 7.5 mmol) and 1 equiv. of N,N-Dimethyl-1,4-phenylendiamin (**An2**) (1.02 mg, 7.5 mmol). Argon-degassed deuterated methanol (0.5 mL, 15 mM) was added to the vial to fully dissolve the compounds and the reaction mixture was transferred into an NMR tube. The NMR tube was charged with argon for 1 min and was sealed. The exchange reaction was monitored *via* ^{1}H-NMR spectroscopy. After the thermodynamic equilibrium state was reached, the NMR tube was irradiated with 430 nm until the photodynamic equilibrium state was obtained.

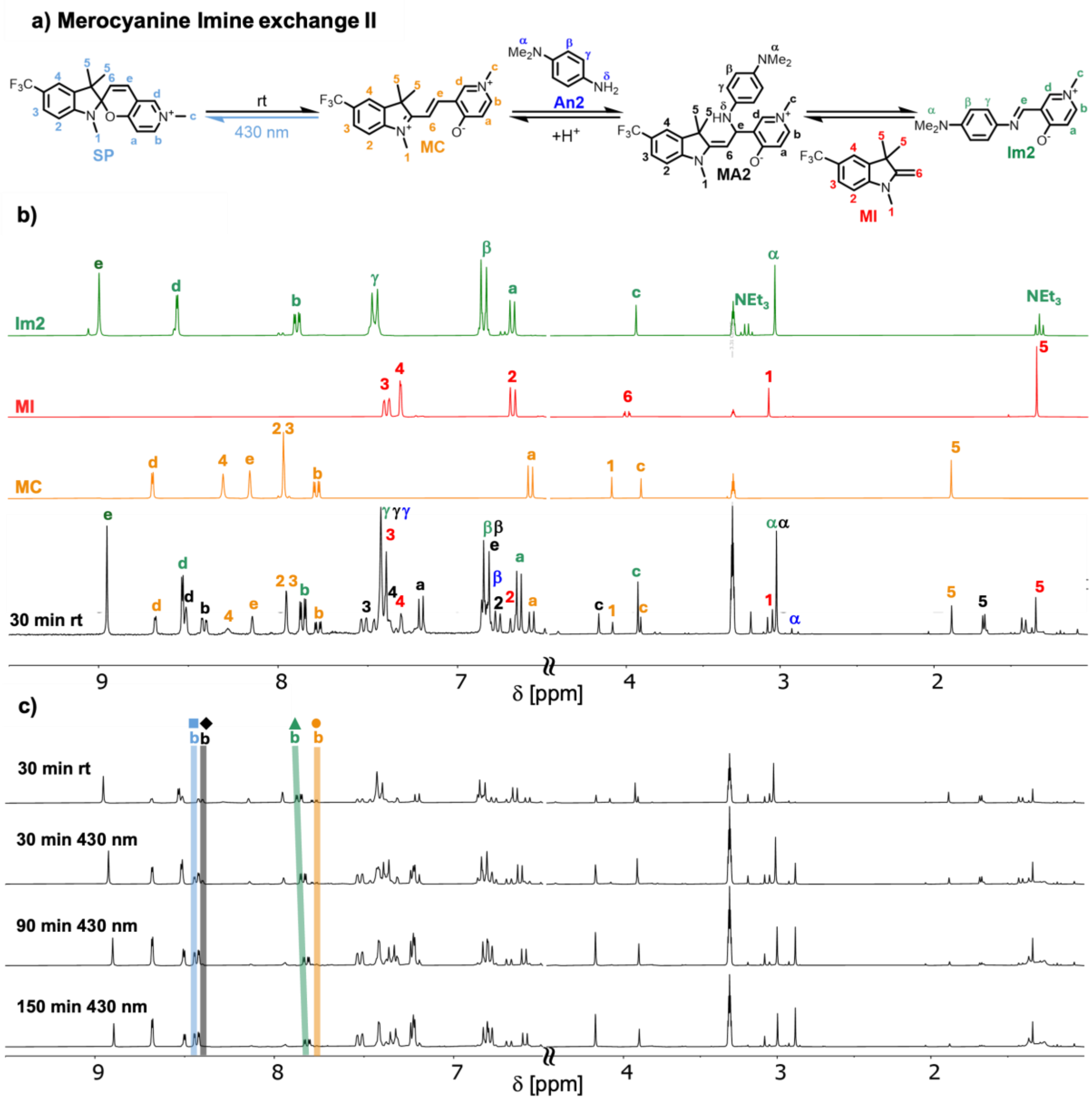

Figure S6. a) Merocyanine-imine exchange via the formation of **MA2** b). Top three spectra: individual ^{1}H-NMR spectra of compounds present in the exchange in CD_3OD, bottom ^{1}H-NMR spectrum of thermodynamic equilibrated merocyanine-imine exchange starting with **MC** and **An2** (c (**MC**) = 15 mM, c (**An2**) = 15 mM) in CD_3OD). b) In situ ^{1}H-NMR monitoring of photodynamic equilibration starting after thermal equilibration (irradiation with 430 nm, Δt = 30, 90, and 150 min in CD_3OD).

2.4 Photochemical ring-closure and thermal ring-opening

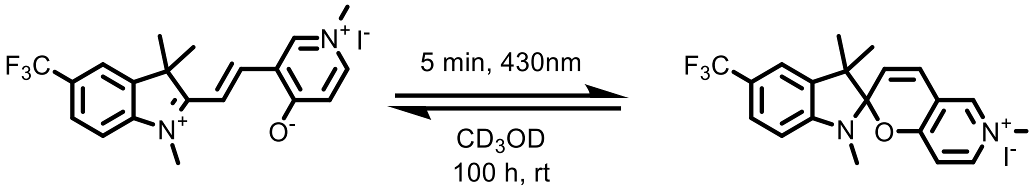

MC SP

A vial was charged with 1-methyl-3-(2-(1,3,3-trimethyl-5-(trifluoromethyl)-3H-indol-1-ium-2-yl)vinyl)pyridin-1-ium-4-olate iodide (**MC**) (3.66 mg, 1 equiv., 7.5 mmol). Deuterated methanol (0.5 mL, 15 mM) was added to the vial and the solution was transferred into an NMR tube. After a ^{1}H-NMR measurement, the sample was irradiated with 430 nm for 5 min to obtain quantitative the closed isomer. The thermal ring-opening isomerization was observed for the first 10 h every 30 min *via* ^{1}H-NMR spectroscopy and additional measurements were performed after 30, 50, and 100 h.

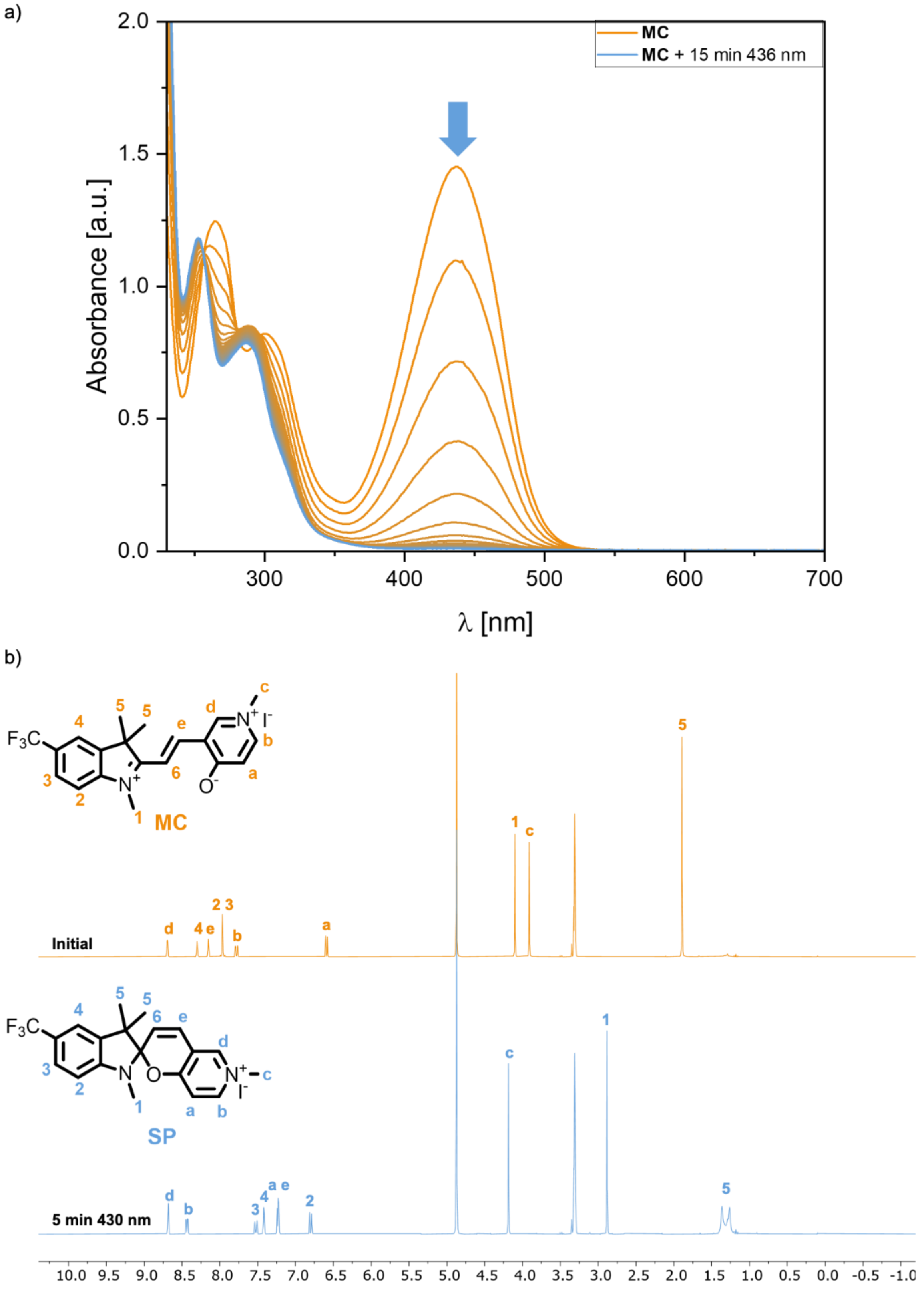

Figure S7. Photoisomerization of **MC**: a) UV/vis absorption spectra upon irradiation with 436 nm were taken every 30 s at 20 °C in 43.5 μM MeOH solution. b) ^{1}H-NMR spectra of **MC** (top) and **SP** (bottom) (*c* (**MC**) = *c* (**SP**) = 15 mM in CD_3OD). **SP** was generated by irradiation of **MC** with 430 nm for ca. 5 min.

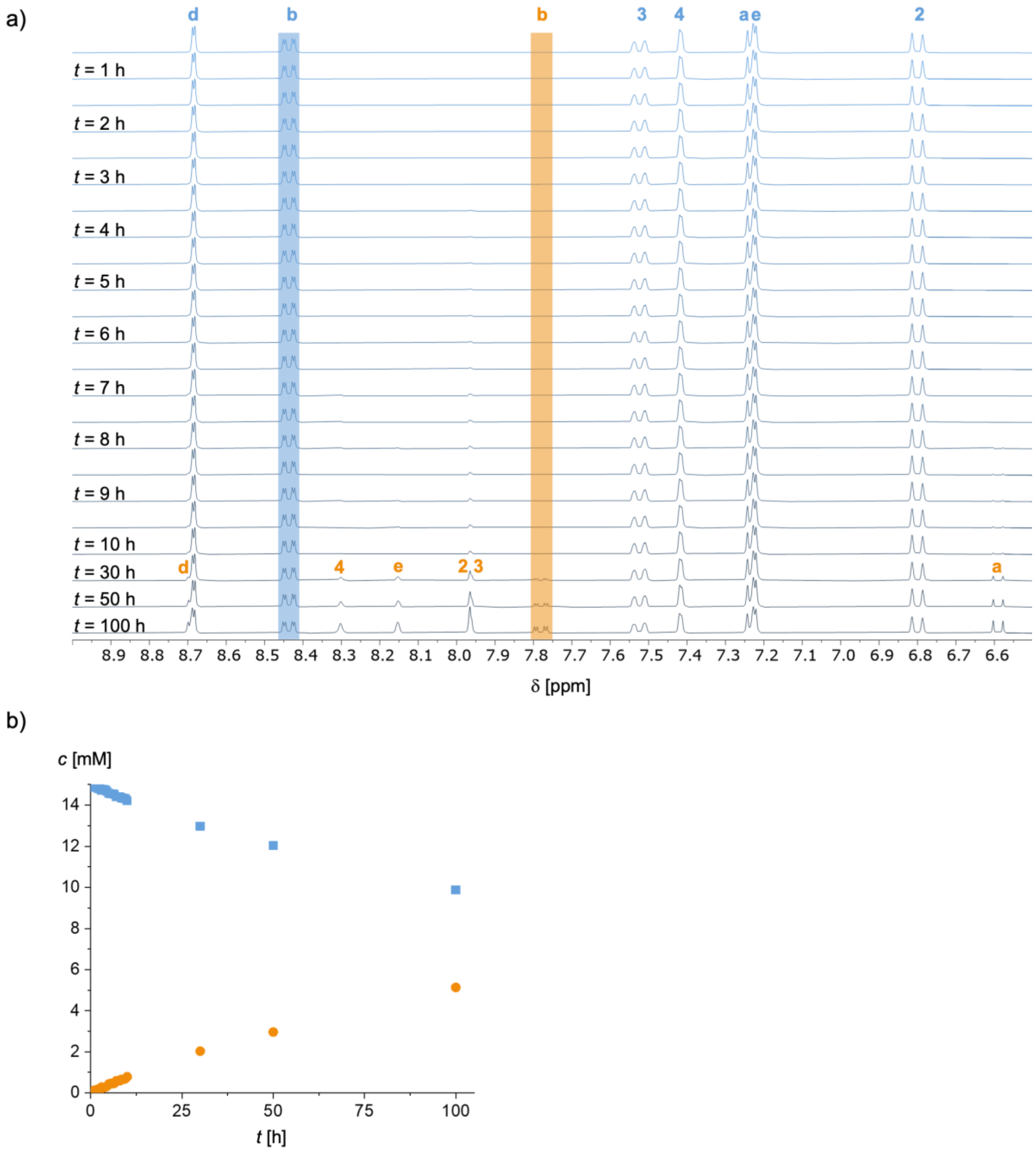

Figure S8. Thermal back isomerization of **SP**: a) In situ ^{1}H-NMR monitoring of **SP** thermal ring-opening at 20 °C in the aromatic region with H_b signals highlighted (first 10 h Δt = 0.5 h afterwards measured at t = 30, 50, and 100 h, c_0(**SP**) = 15 mM in CD_3OD). b) Concentration profile of **SP** and **MC** as determined by integration of the H_b signals at 8.44 ppm (**SP**) and 7.78 ppm (**MC**).

3. Appendix

3.1 NMR Spectra

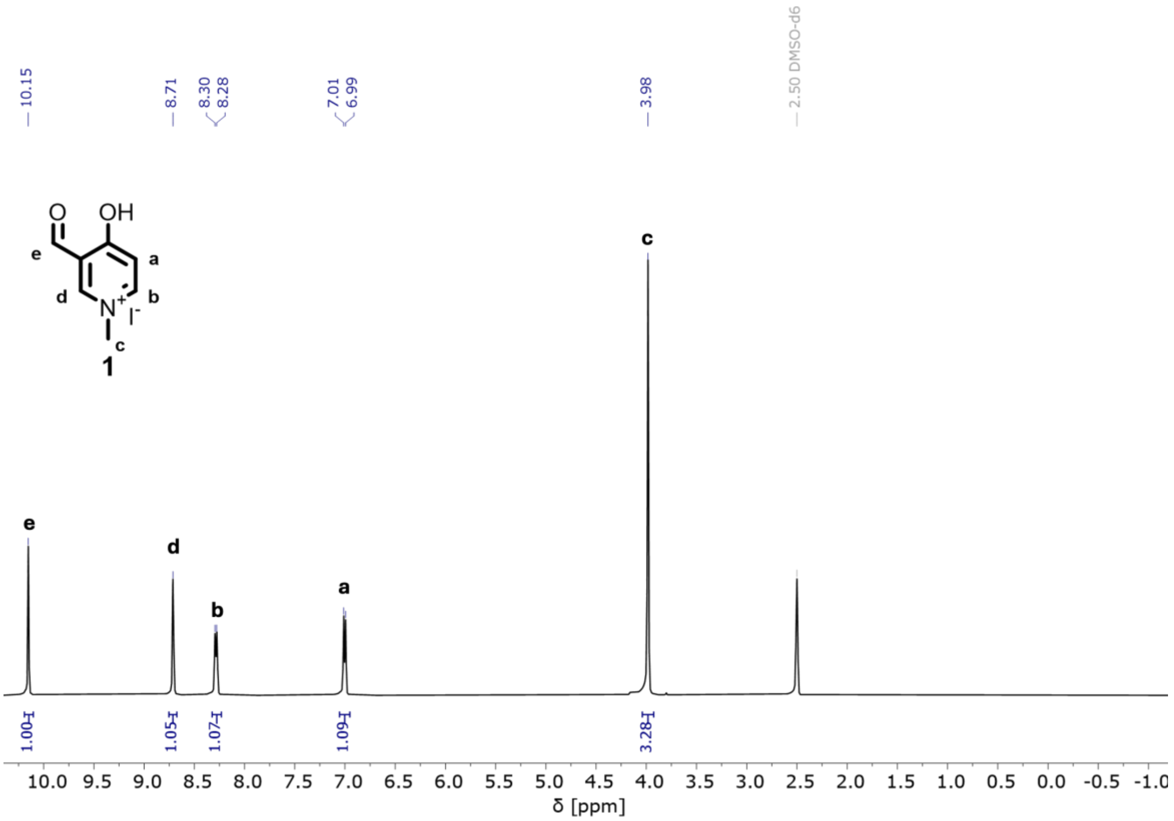

Figure S9. ^{1}H-NMR spectrum of **1** measured in DMSO-d_6 at 298 K.

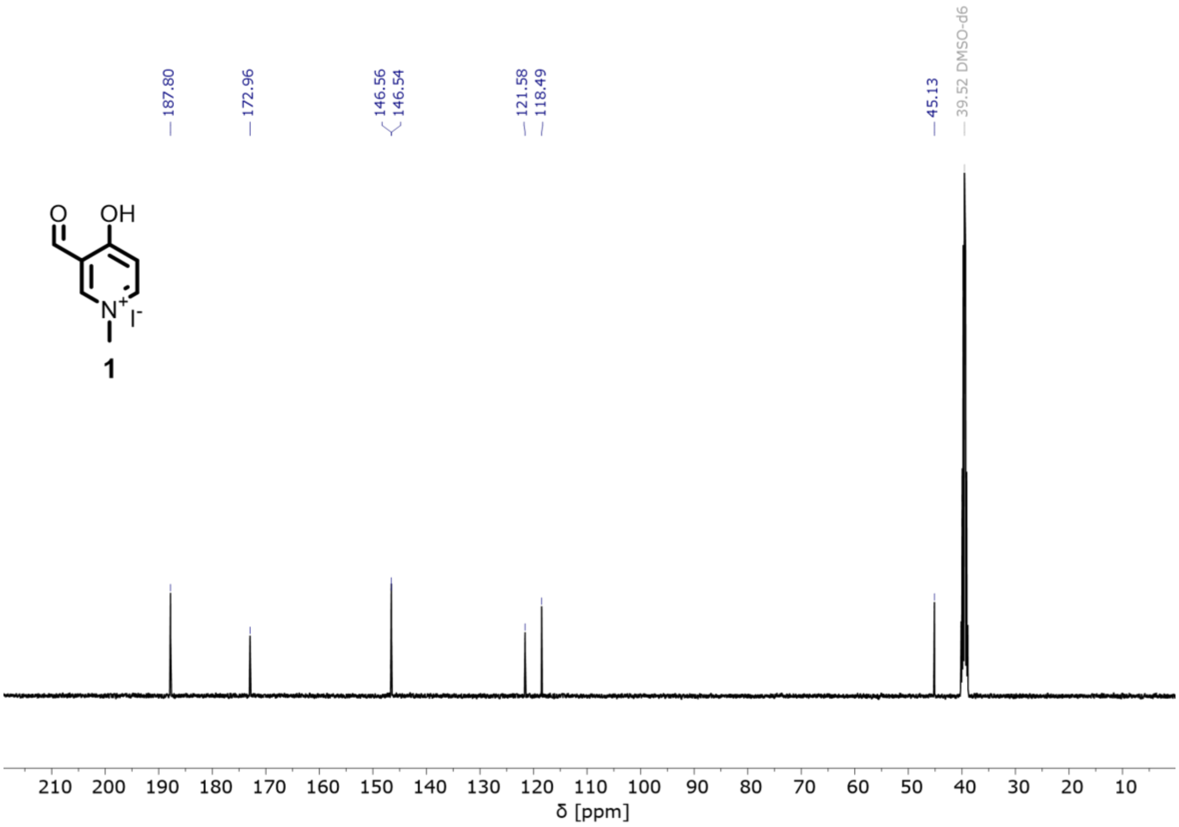

Figure S10. ^{13}C-NMR spectrum of **1** measured in DMSO-d_6 at 298 K.

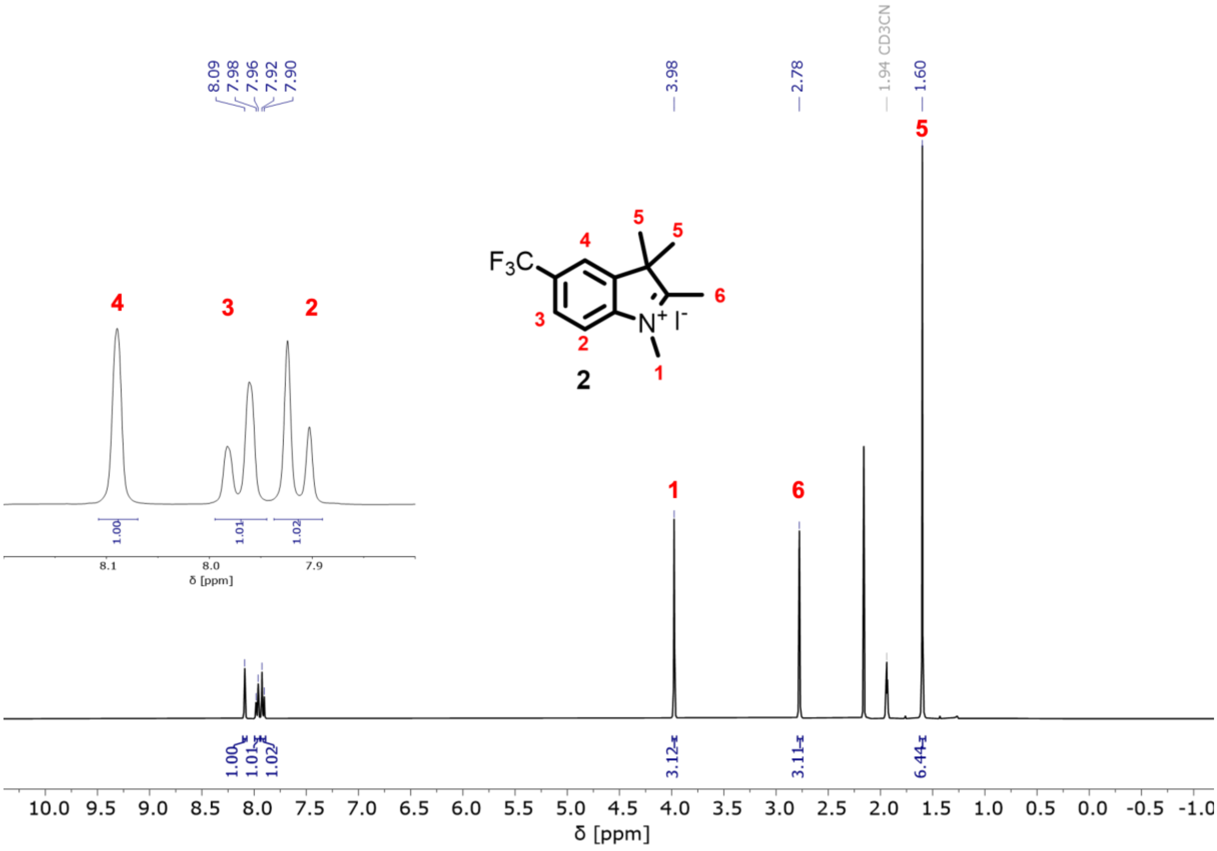

Figure S11. ^{1}H-NMR spectrum of **2** measured in CD_3CN at 298 K.

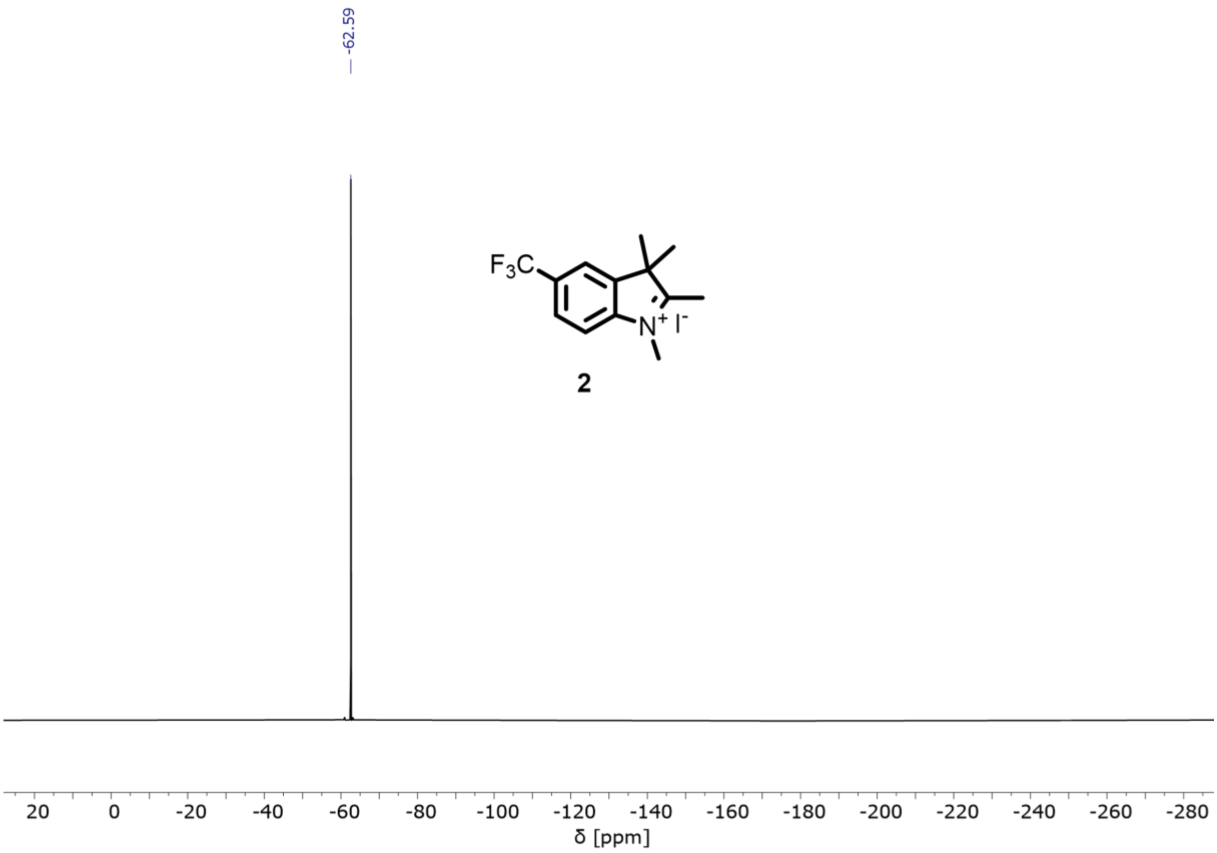

Figure S12. ^{19}F-NMR spectrum of **2** measured in CD_3CN at 298 K.

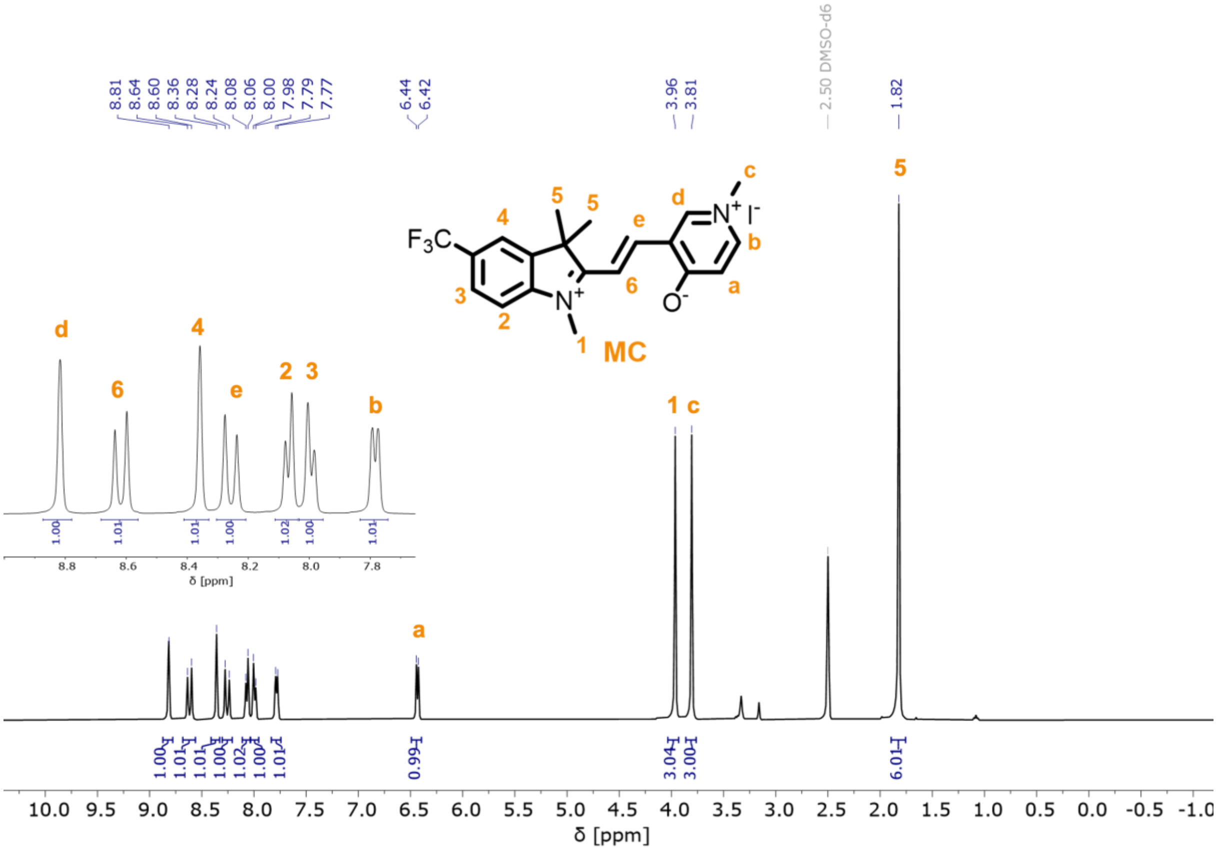

Figure S13. ^{1}H-NMR spectrum of **MC** measured in DMSO-d_6 at 298 K.

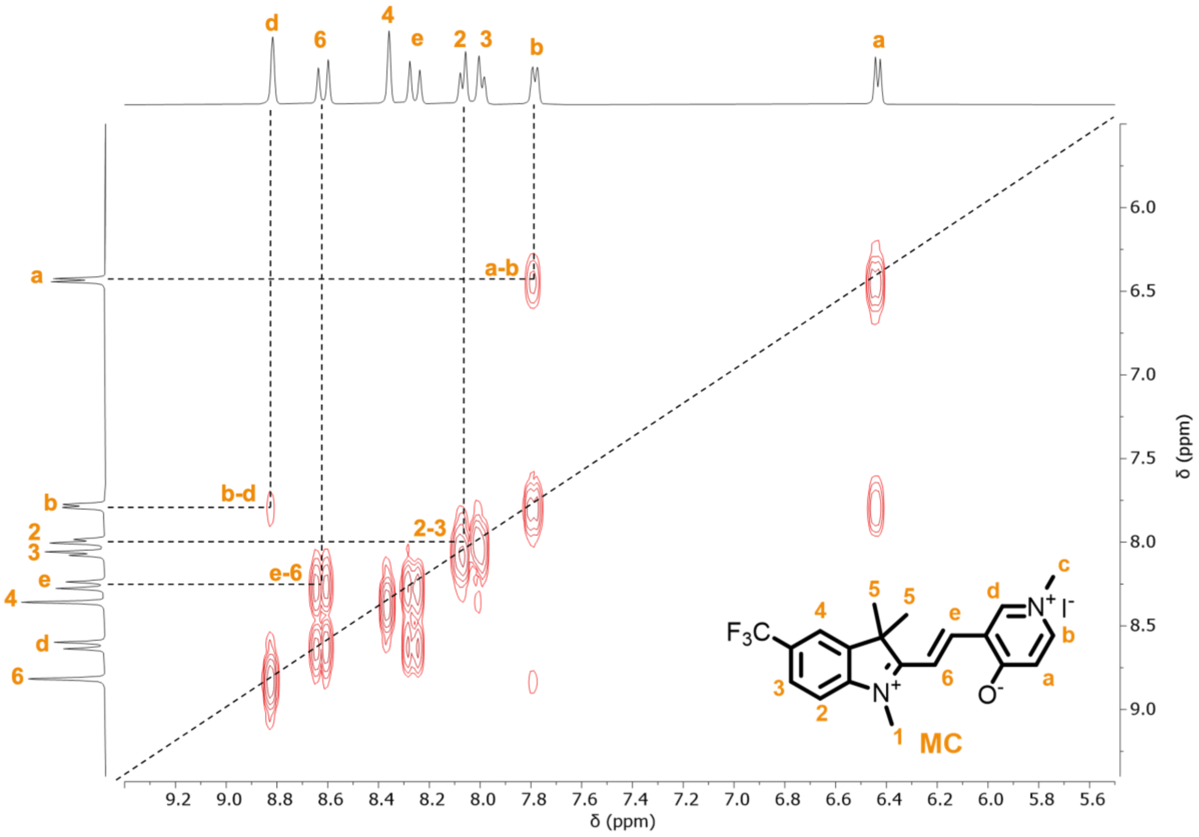

Figure S14. COSY-NMR spectrum of **MC** measured in DMSO-d_6 at 298 K.

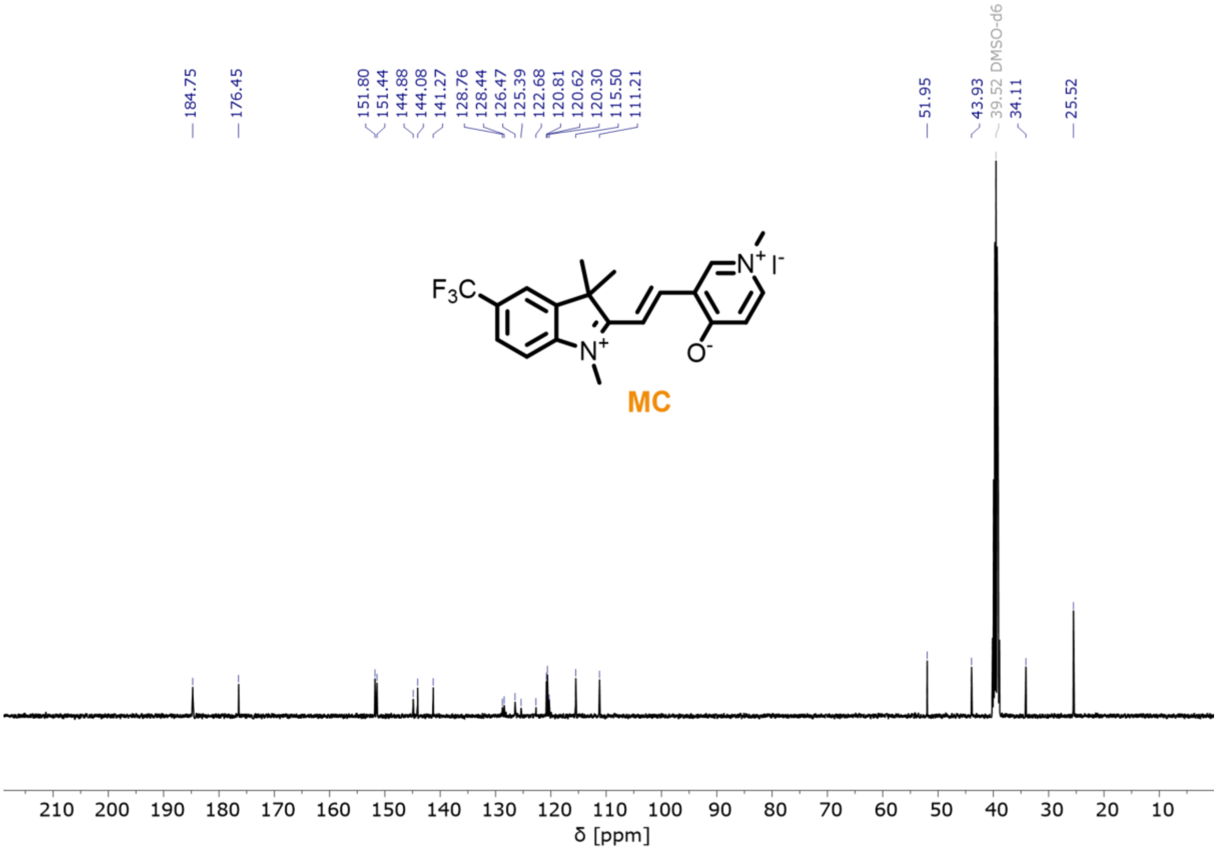

Figure S15. ^{13}C-NMR spectrum of **MC** measured in DMSO-d_6 at 298 K.

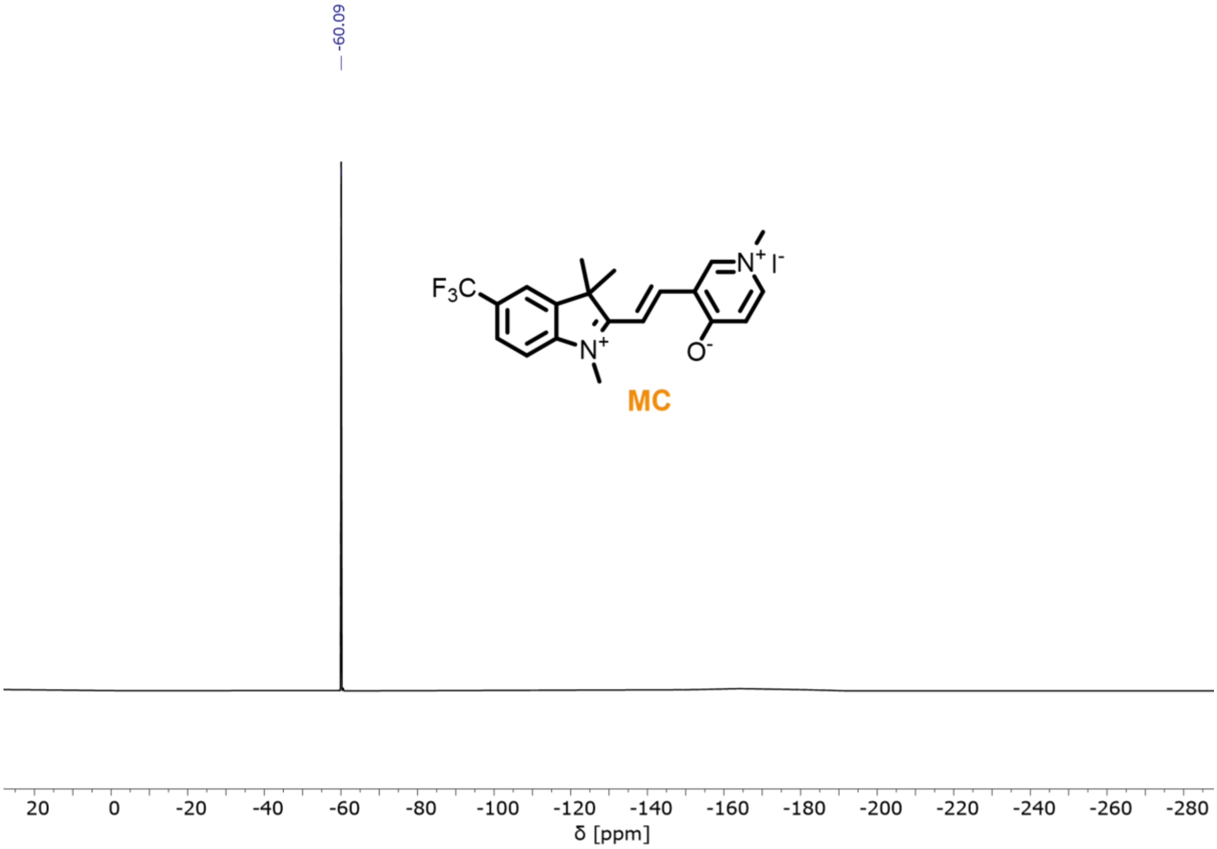

Figure S16. ^{19}F-NMR spectrum of **MC** measured in DMSO-d_6 at 298 K.

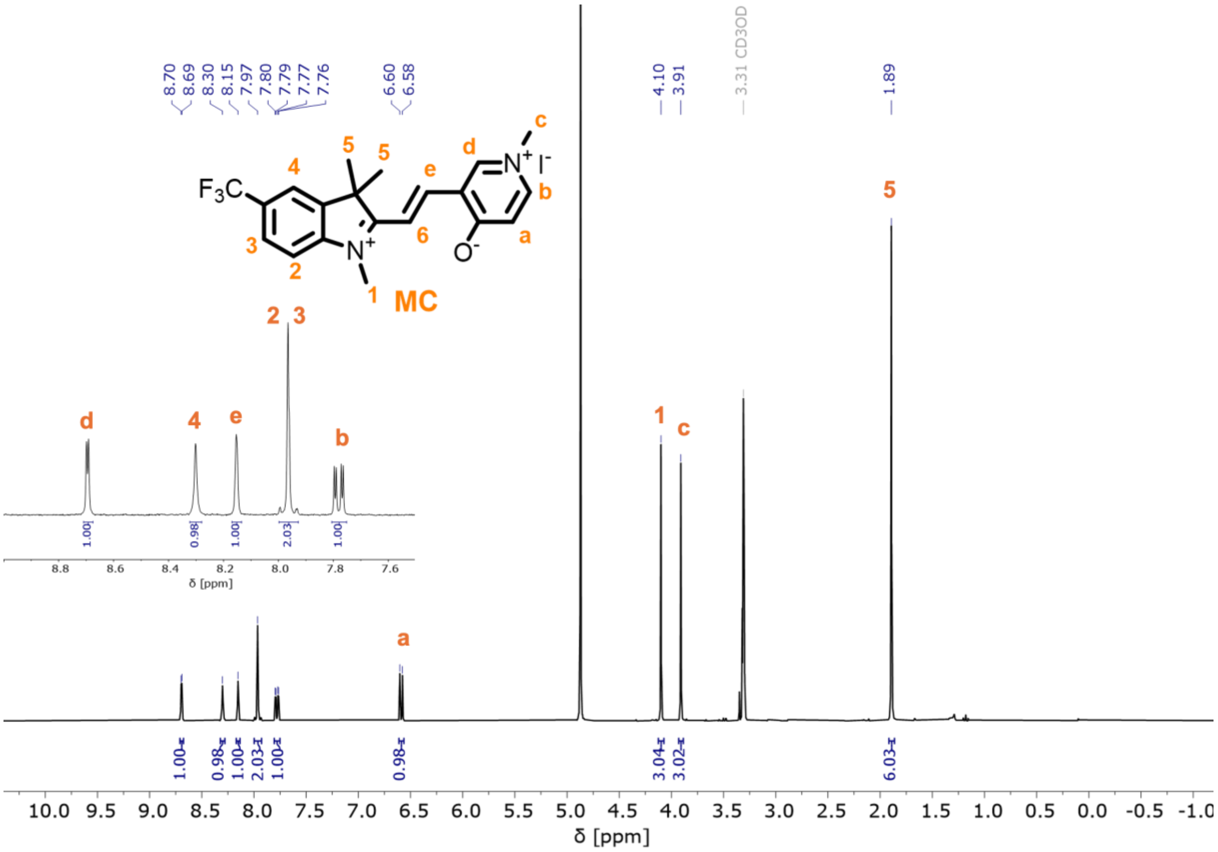

Figure S17. ^{1}H-NMR spectrum of **MC** measured in CD_3OD at 298 K.

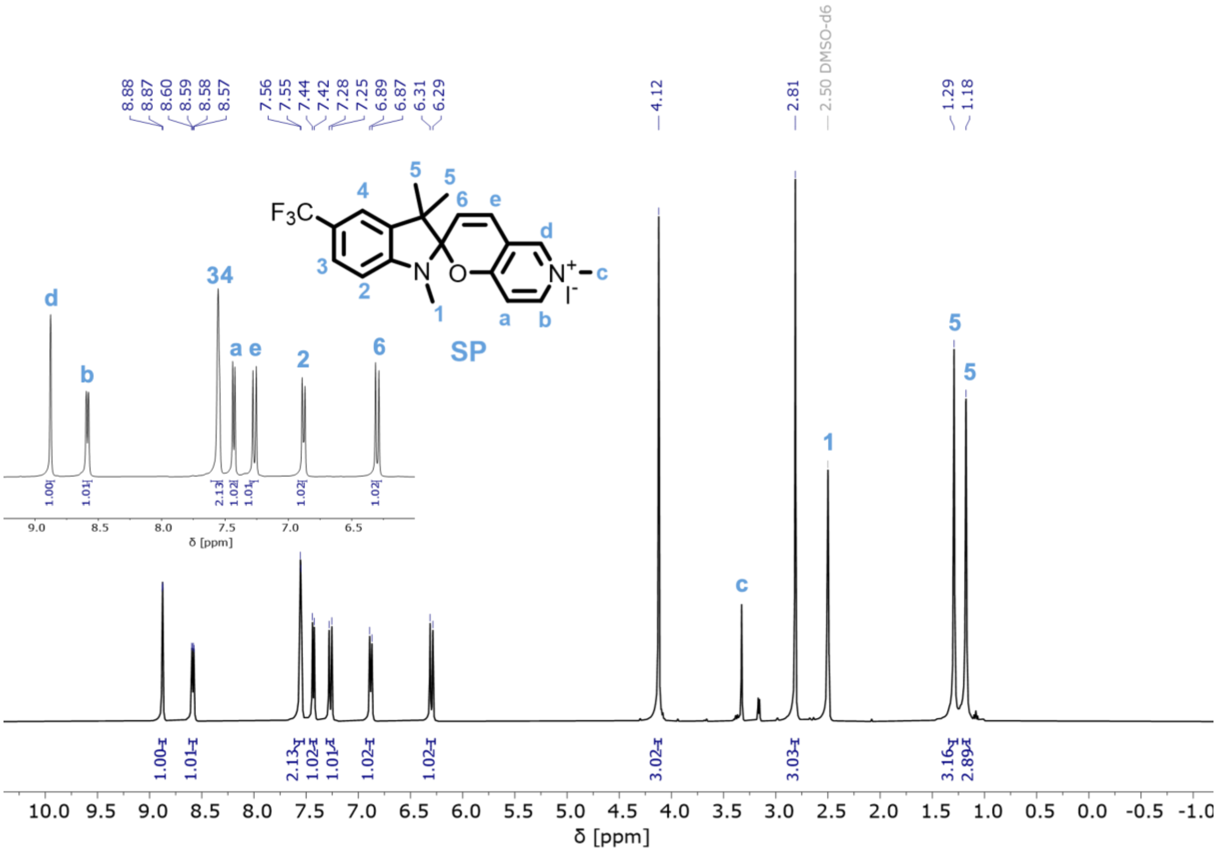

Figure S18. ^{1}H-NMR spectrum of **SP** measured in DMSO-d_6 at 298 K.

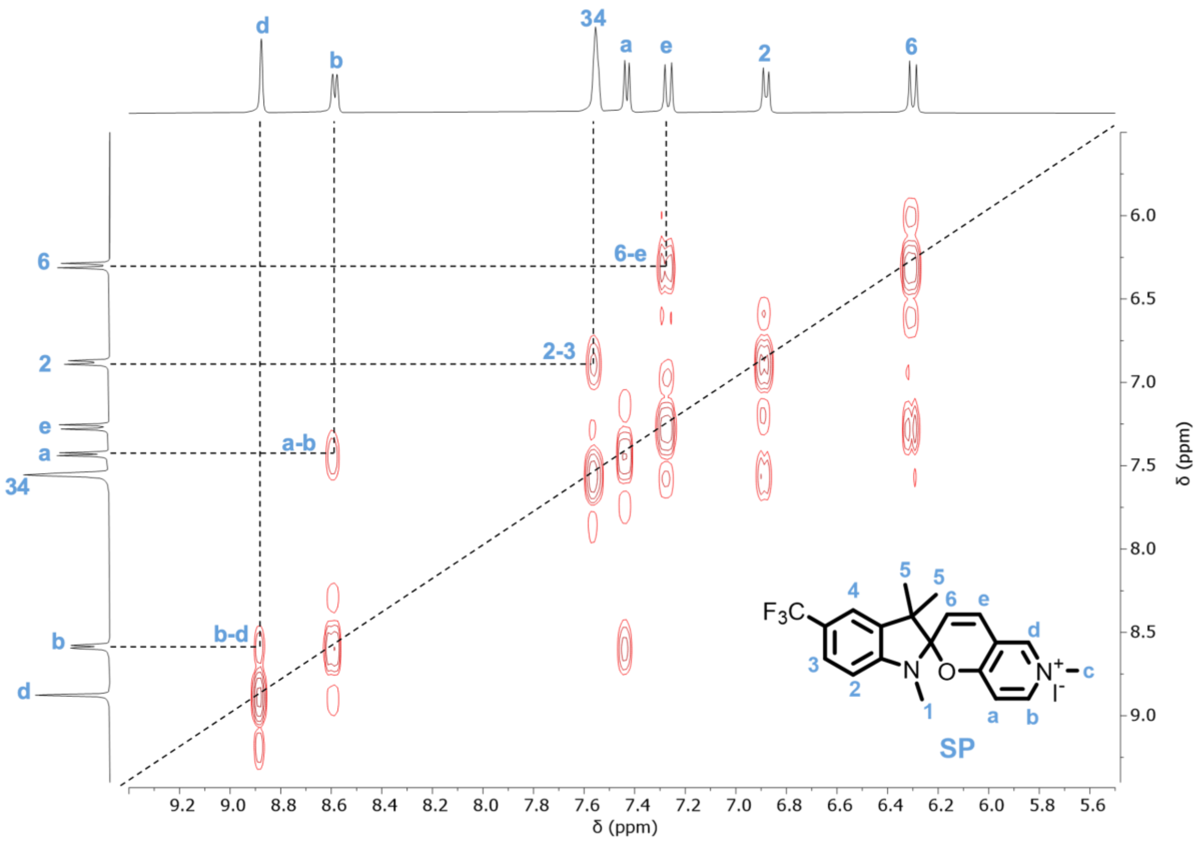

Figure S19. COSY-NMR spectrum of **SP** measured in DMSO-d_6 at 298 K.

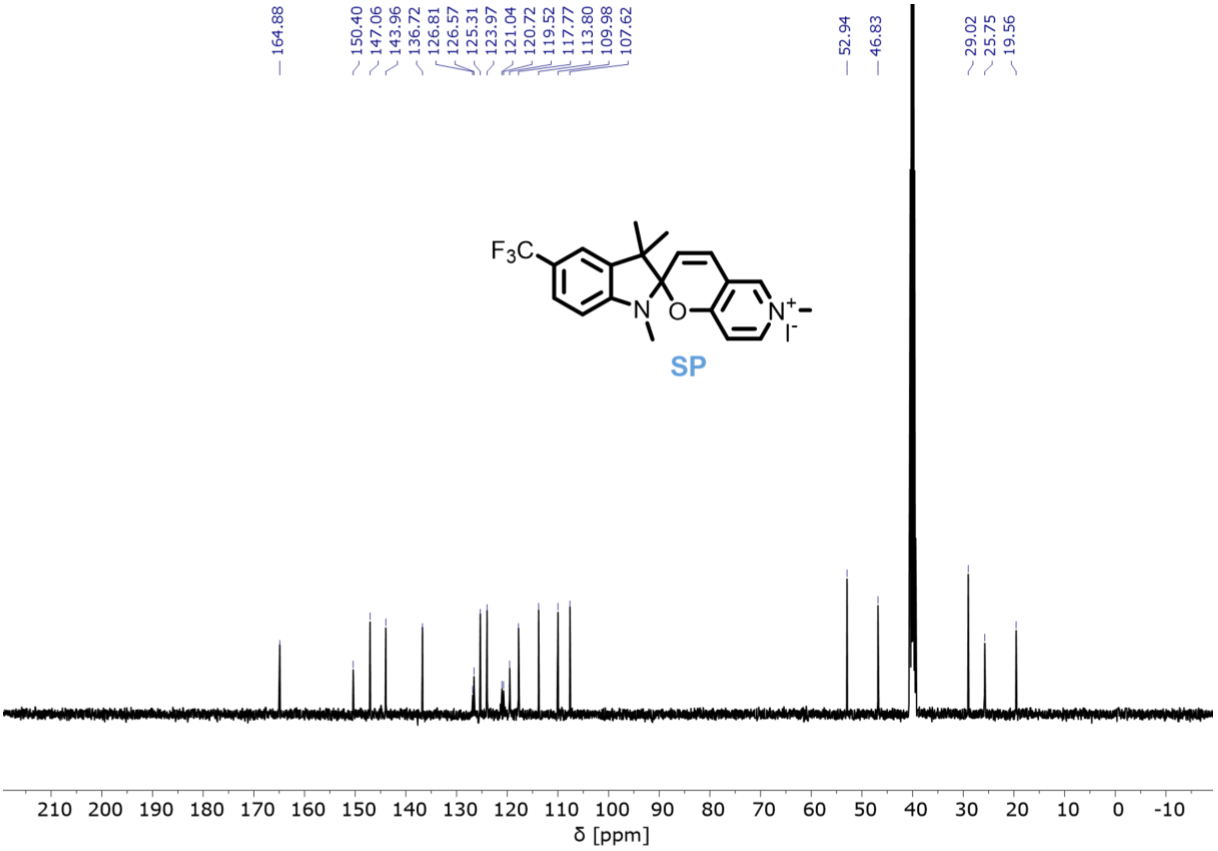

Figure S20. ^{13}C-NMR spectrum of **SP** measured in DMSO-d_6 at 298 K.

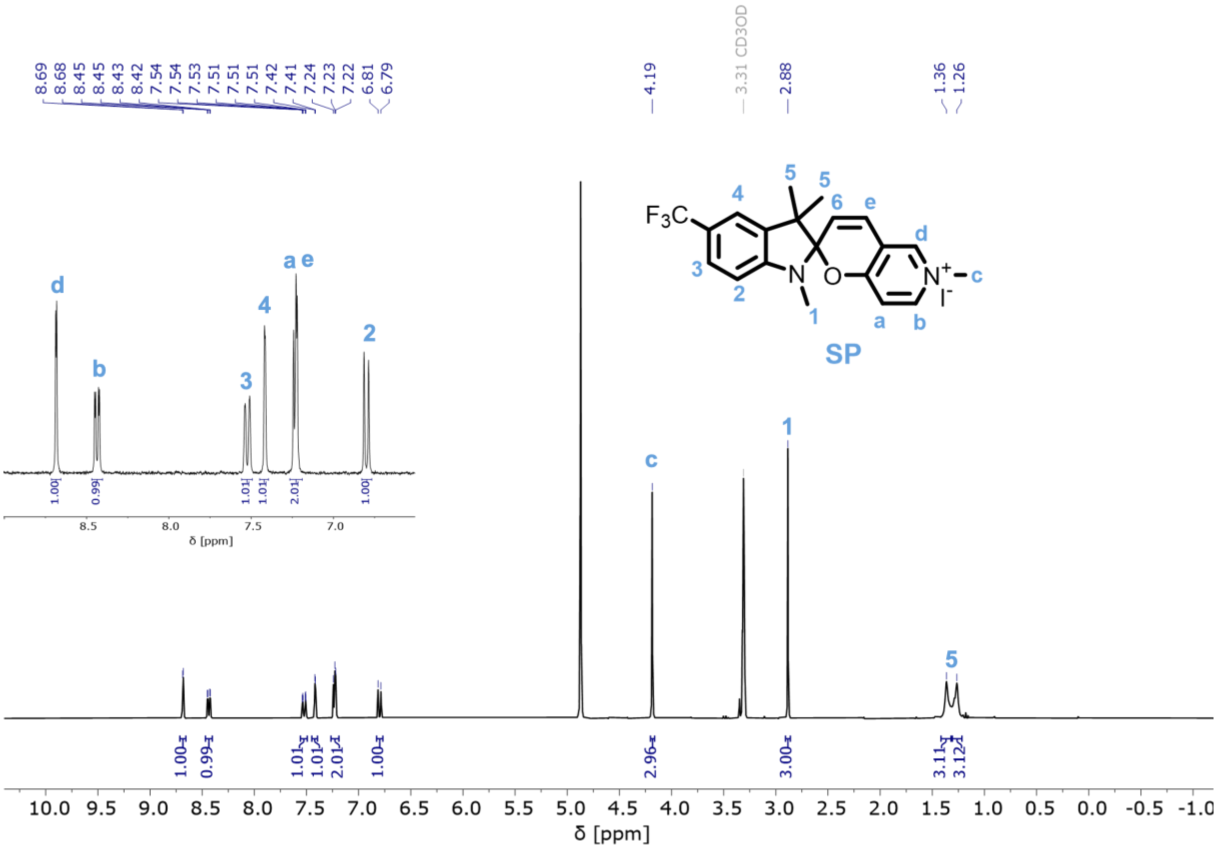

Figure S21. ^{1}H-NMR spectrum of **SP** measured in CD_3OD at 298 K.

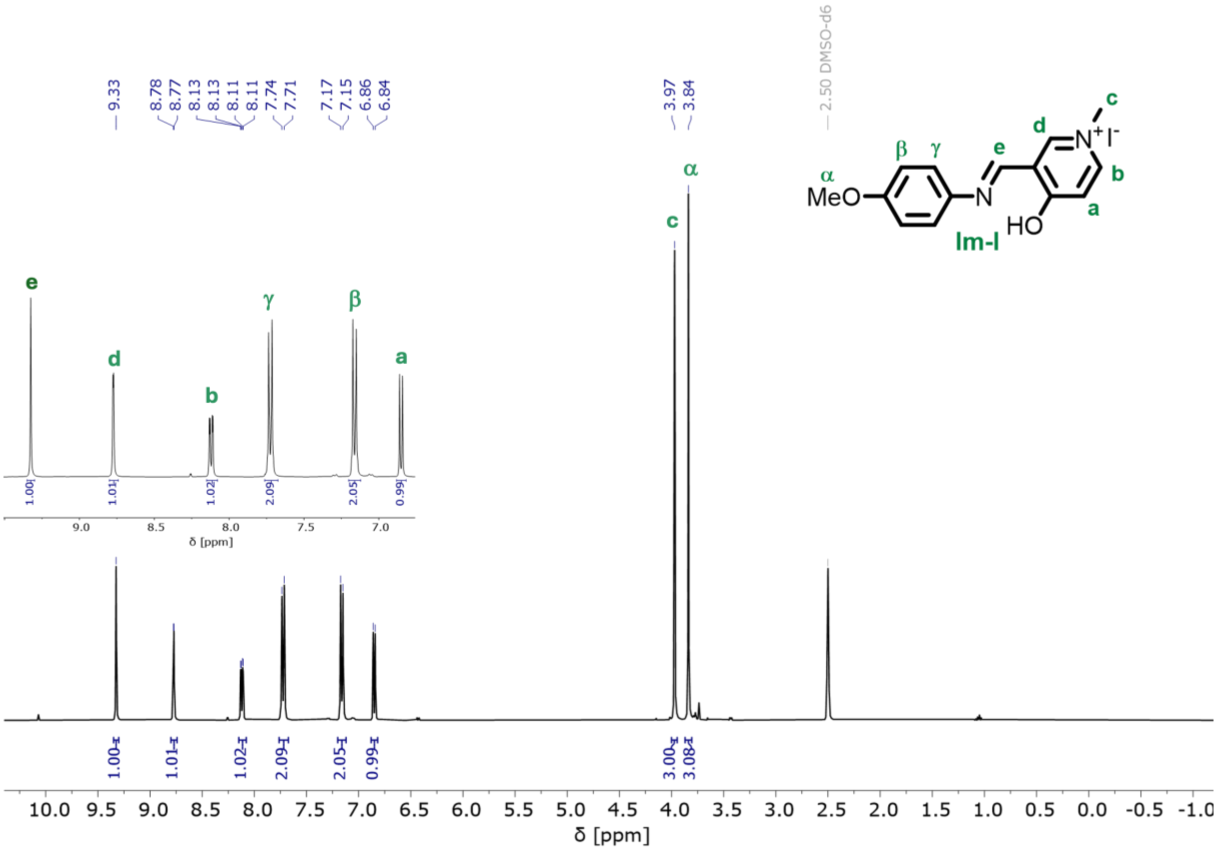

Figure S22. ^{1}H-NMR spectrum of **Im-I** measured in DMSO-d_6 at 298 K.

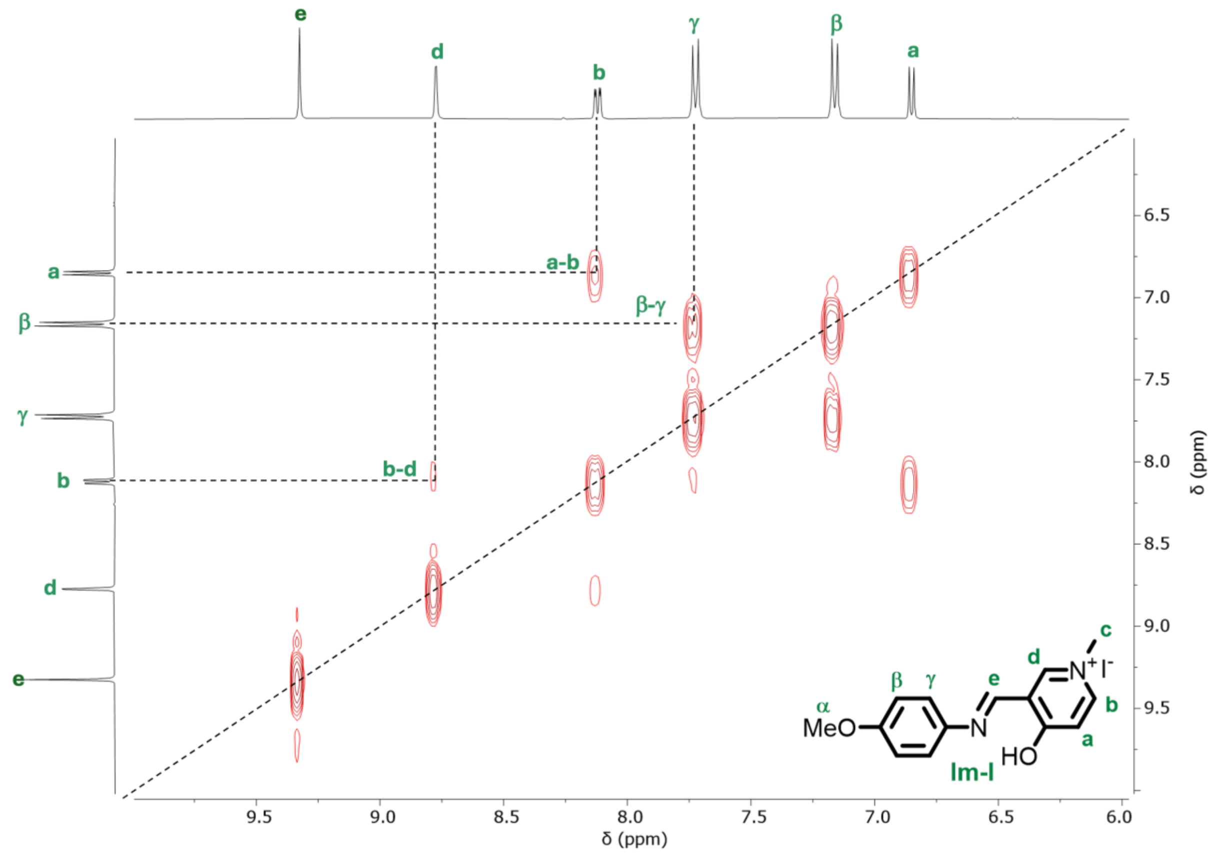

Figure S23. COSY-NMR spectrum of **Im-I** measured in DMSO-d_6 at 298 K.

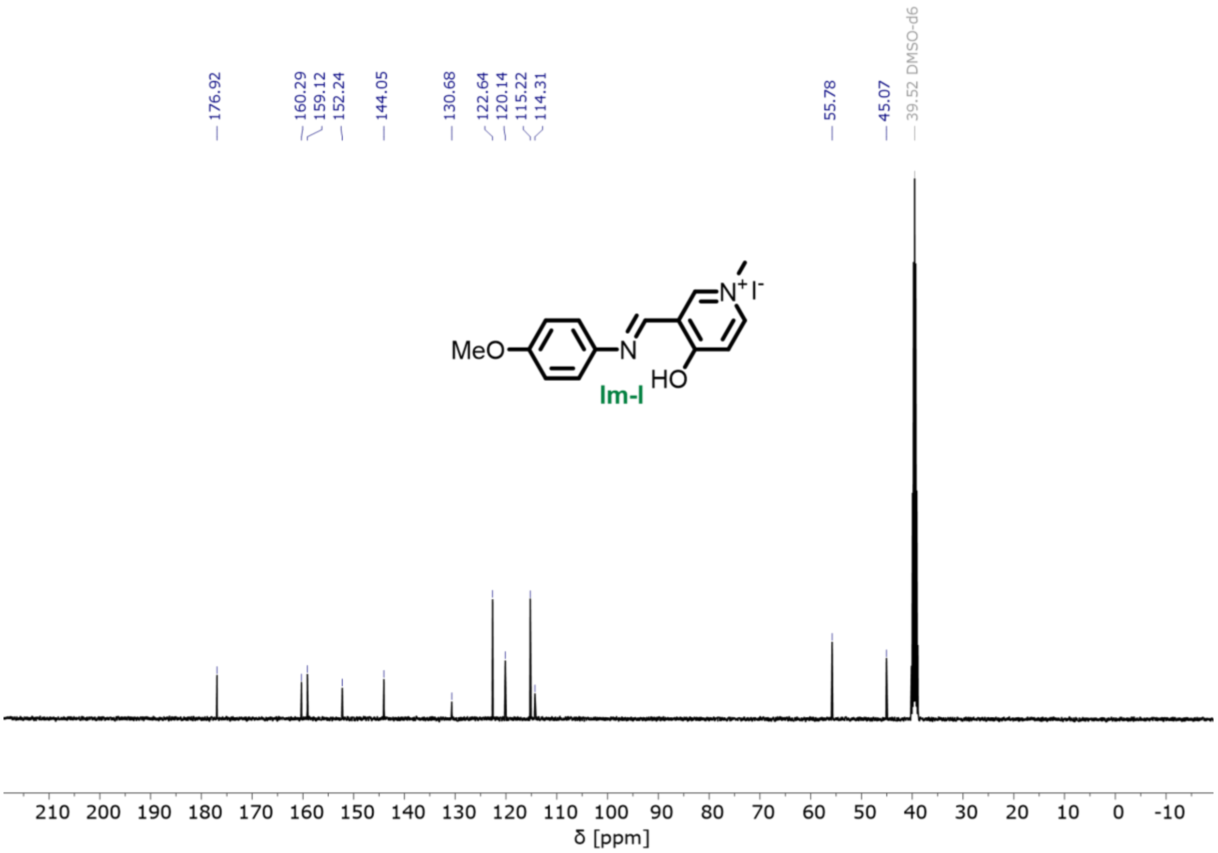

Figure S24. ^{13}C-NMR spectrum of **Im-I** measured in DMSO-d_6 at 298 K.

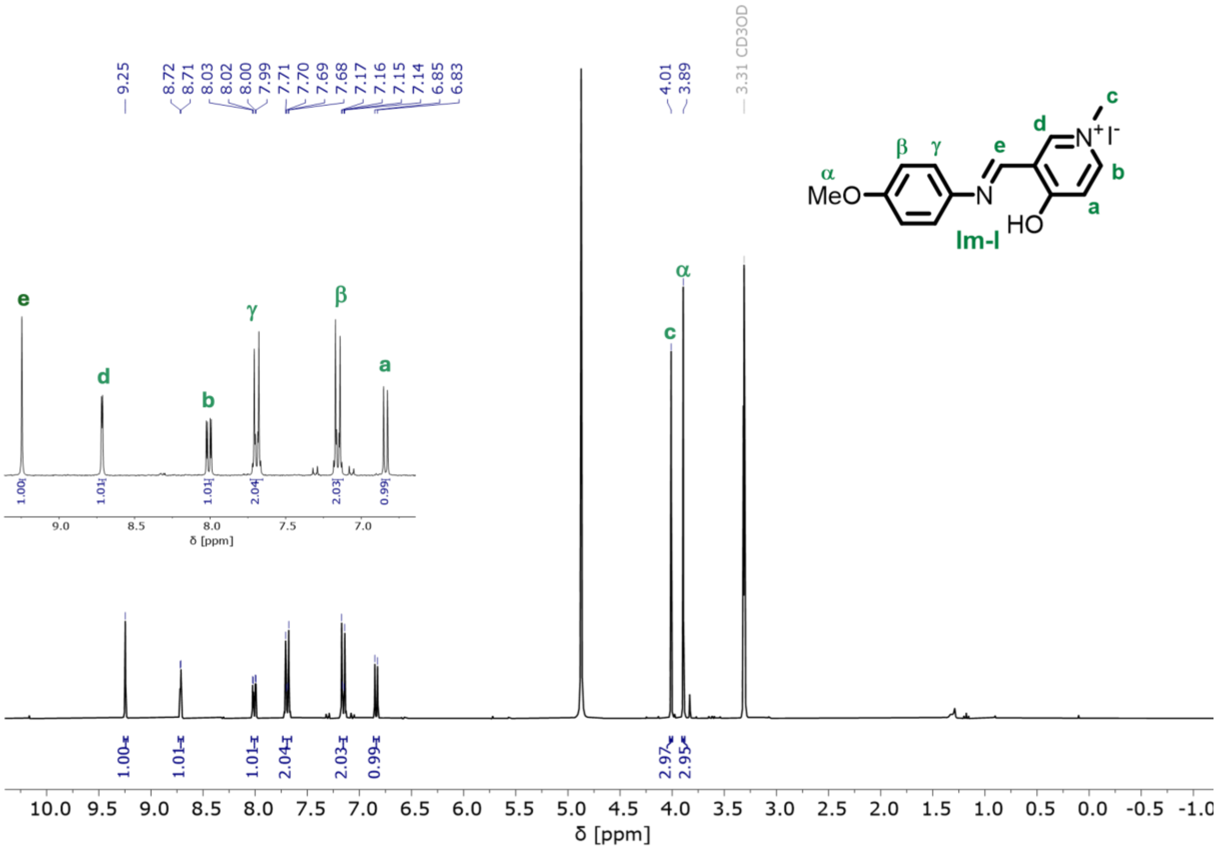

Figure S25. ^{1}H-NMR spectrum of **Im-I** measured in CD_3OD at 298 K.

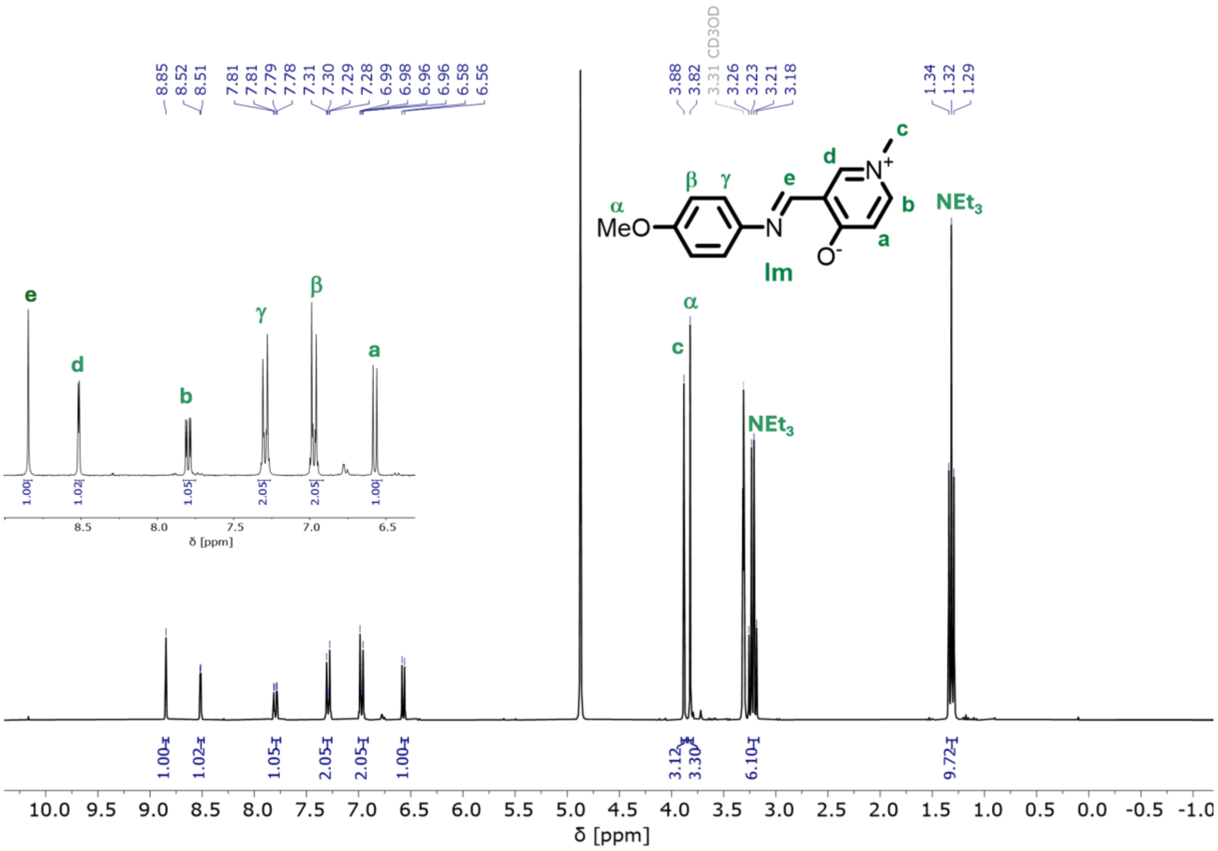

Figure S26. ^{1}H-NMR spectrum of **Im** measured in CD_3OD at 298 K.

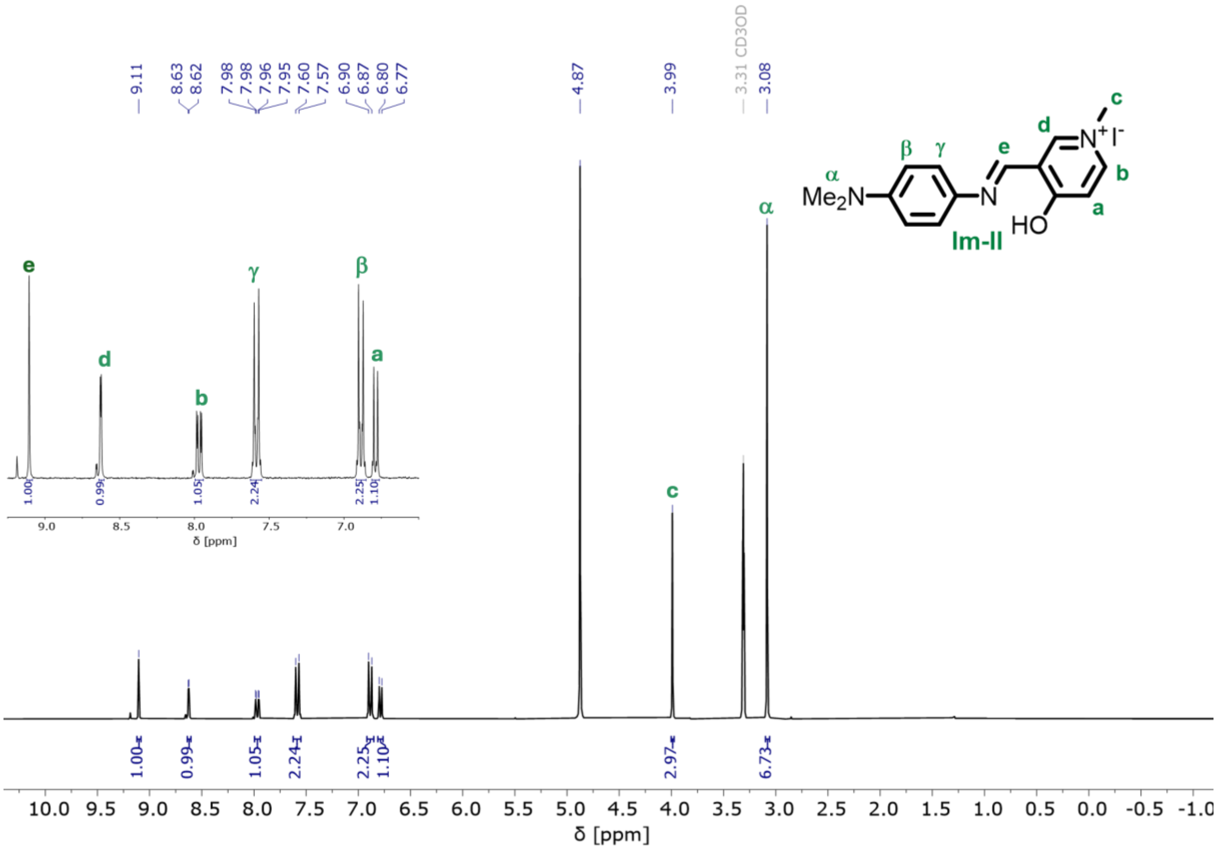

Figure S27. ^{1}H-NMR spectrum of **Im-II** measured in CD_3OD at 298 K.

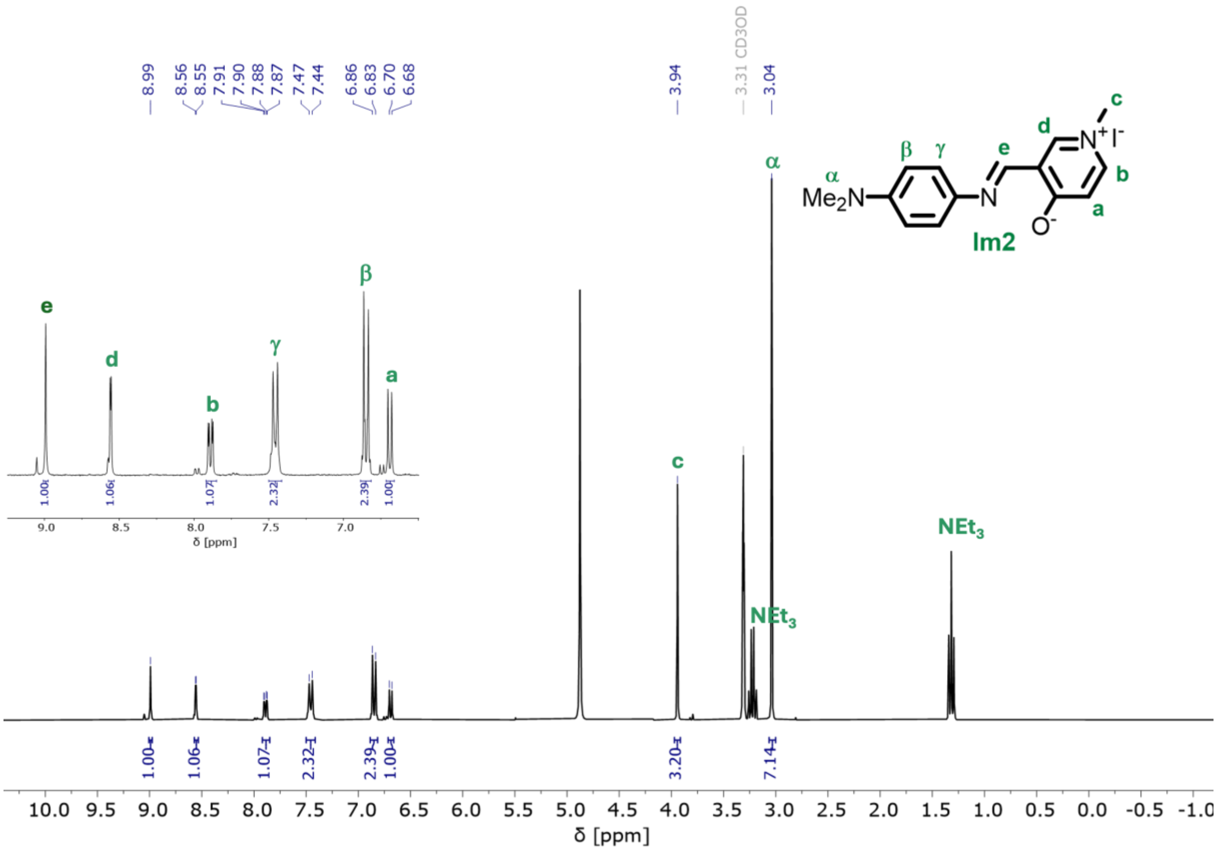

Figure S28. ^{1}H-NMR spectrum of **Im2** measured in CD_3OD at 298 K.

4. Reference

(1) Drichel, A.; Garmshausen, Y.; Hecht, S. Dynamic Covalent Spiropyran Exchange for Rapid Structural Diversification. *Angew. Chem. Int. Ed*. **2025**, *64*, e20241953.
